# DPEP1 is a direct target of miR-193a-5p and promotes hepatoblastoma progression by PI3K/Akt/mTOR pathway

**DOI:** 10.1038/s41419-019-1943-0

**Published:** 2019-09-20

**Authors:** Xichun Cui, Xin Liu, Qicai Han, Jianming Zhu, Jianhao Li, Zhigang Ren, Liwen Liu, Yanbing Luo, Zhifang Wang, Dandan Zhang, Yingzhong Fan, Da Zhang, Gang Dong

**Affiliations:** 1 Department of Pediatric Surgery, The First Affiliated Hospital of Zhengzhou University, 450052 Zhengzhou, China; 2 Precision Medicine Center, The First Affiliated Hospital of Zhengzhou University, 450052 Zhengzhou, China; 3 Department of Pathology, The First Affiliated Hospital of Zhengzhou University, 450052 Zhengzhou, China; 4 Department of Ultrasonography, The First Affiliated Hospital of Zhengzhou University, 450052 Zhengzhou, China

**Keywords:** Paediatric cancer, Tumour biomarkers

## Abstract

Hepatoblastoma (HB) is the most common hepatic neoplasm in childhood and the therapeutic outcomes remain undesirable due to its recurrence and metastasis. Increasing evidence shows that dipeptidase 1 (DPEP1) has pivotal function in tumorigenesis in multiple tumors. However, the expression pattern, biological function, and underlying mechanism of DPEP1 in HB have not been reported. Here we showed that DPEP1 was significantly upregulated and was associated with poor prognosis in HB patients. In vitro and in vivo assays indicated that silencing DPEP1 significantly suppressed HB cell proliferation, migration, and invasion, while DPEP1 overexpression exhibited the opposite effect. In addition, we identified that DPEP1 was a direct target of microRNA-193a-5p (miR-193a-5p). Functional experiments demonstrated that overexpression of miR-193a-5p significantly inhibited cell proliferation and invasion of HB cells, while the inhibitory effect could be reversed by DPEP1 overexpression. Moreover, miR-193a-5p was decreased in HB tumor tissues and associated with a poor clinical prognosis. Mechanistically, our results indicated that the miR-193a-5p/DPEP1 axis participated to the progression of HB via regulating the PI3K/Akt/mTOR (phosphatidylinositol-3-kinase/Akt/mammalian target of rapamycin) signaling. In conclusion, our findings suggest that the miR-193a-5p /DPEP1 axis might be a good prognostic predictor and therapeutic target in HB.

## Introduction

Hepatoblastoma (HB) is one of the most highly invasive malignant carcinoma in children, which accounts for ~50% of pediatric liver cancers^1,2^. However, about 20% of those patients already have metastasis at the first diagnosis^3^. Despite regional and local control of HB have improved owing to the application of adjuvant chemotherapy, surgical resection, and liver transplantation, the prognosis for patients in advanced HB stages remains very poor^4,5^. Thus, it is particularly important to identify valid biomarkers for early diagnosis and treatment of HB.

Dipeptidase 1 (DPEP1) is a zinc-dependent metalloproteinase, and participates in the metabolism of glutathione and other similar compounds by dipeptide hydrolysis^6^. Recently, more attention has been attracted to the role of DPEP1 in human malignancies. DPEP1 is frequently dysregulated in many tumors, including colorectal cancer, colon cancer, and breast cancer^7–9^. However, the expression pattern and function of DPEP1 in HB is not clear.

MicroRNAs (miRNAs) are a class of small noncoding RNAs with ~22 nucleotide that can regulate the targeted gene expression by binding to the 3′-untranslated region (UTR) of their target messenger RNAs (mRNAs)^10^. Increasing evidence has found that miRNAs play a critical role in the tumorigenesis of most human malignancies, including HB^11,12^. Various miRNAs have been studied in the tumorigenesis and metastasis of HB, such as miR-21, miR-17, miR-492, miR-124, and miR-206^12–15^. MiR-193a-5p has been investigated as a tumor suppressor in multiple cancers^16–19^. However, the specific function of miR-193a-5p in HB tumorigenesis and progression is unknown.

In this current study, we found the upregulated expression of DPEP1 in HB patient tumor tissues and HB cell lines. Silencing DPEP1 inhibited HB cell proliferation, migration, and invasion in vitro, and inhibited HB tumor development in vivo. Further, we identified that miR-193a-5p directly targeted DPEP1 by binding to its 3′-UTR. Cell proliferation and invasion capacity were inhibited by miR-193a-5p overexpression, which could be reversed by the overexpression of DPEP1. We also demonstrated that high expression of DPEP1 or low expression of miR-193a-5p was remarkably related to poor prognosis in HB patients. Moreover, we identified that miR-193a-5p/DPEP1 axis was critical in regulating PI3K/Akt/mTOR (phosphatidylinositol-3-kinase/Akt/mammalian target of rapamycin) signaling. Taken together, our results indicate miR-193a-5p/DPEP1 as a novel regulatory axis in HB, which could be utilized as an effective therapeutic target in HB.

## Results

### DPEP1 is upregulated in HB tissues and high DPEP1 expression correlates with poor prognosis in HB patients

**Fig. 1:**
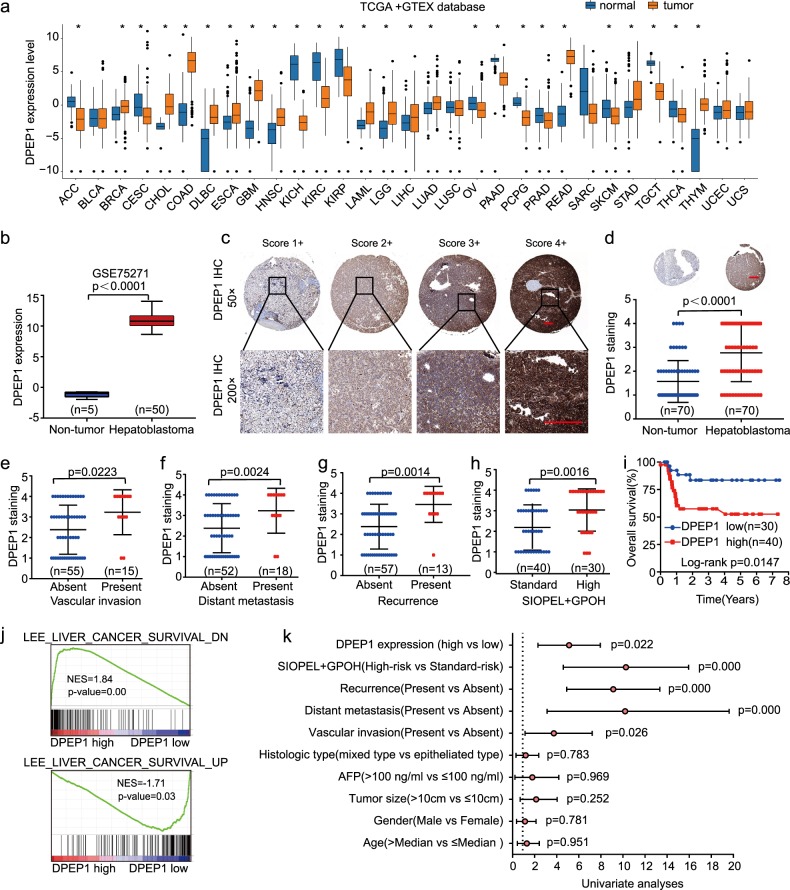
DPEP1 is upregulated in HB tissues and high DPEP1 expression correlates with poor prognosis in HB patients. **a** Bioinformatics analysis of DPEP1 expression in TCGA and GTEX database. **b** Bioinformatics analysis of DPEP1 mRNA expression in the Gene Expression Omnibus (GEO) database (GSE75271). **c** Immunohistochemical (IHC) staining of DPEP1 on tissue microarray (TMA) and the scoring system based on the IHC staining intensity of DPEP1. Scale bars, 100 μm. **d** Representative IHC staining of DPEP1 and the distribution of DPEP1 staining intensity in HB tissues and non-tumor control tissues. Scale bars, 200 μm. **e–h** The correlation between the expression levels of DPEP1 with vascular invasion (**e**), tumor distant metastasis (**f**), cancer recurrence (**g**), or SIOPEL + GPOH risk stratification (**h**). **i** Kaplan–Meier survival analysis of overall survival rate between HB patients with low or high DPEP1 expression. **j** The gene set enrichment analysis (GSEA) analysis the correlation between liver cancer survival gene set and the expression levels of DPEP1. **k** Forest plot depicting correlations between the indicated clinical criteria and the expression level of DPEP1. *P < 0.05

To explore DPEP1 expression pattern in different cancers, we analyzed the expression levels of DPEP1 mRNA in various types of cancers using The Cancer Genome Atlas (TCGA) and Genotype-Tissue Expression (GTEX) database. The results suggested that DPEP1 was highly expressed in most cancers (Fig. 1a). Meanwhile, the analysis of Gene Expression Omnibus (GEO) database (GSE75271) revealed that DPEP1 was drastically upregulated in HB tissues in comparison with that in non-tumor control tissues at the mRNA level (Fig. 1b). To further confirm the expression of DPEP1 protein in HB, immunohistochemistry (IHC) was performed on HB tissue microarrays (TMAs). We used a scoring system based on the IHC staining intensity of DPEP1 (Fig. 1c). Consistently, DPEP1 expression was markedly enhanced in HB tissues in comparison with that in normal control tissues (Fig. 1d).

Furthermore, we found that high DPEP1 expression was associated with vascular invasion, distant metastasis, recurrence, and SIOPEL + GPOH (International Society of Pediatric Oncology – Liver Tumor Strategy Group+German Society for Pediatric Oncology and Hematology) risk stratification (Fig. 1e–h and Table 1). Kaplan–Meier analysis also demonstrated that overall survival (OS) was markedly reduced in HB-afflicted children with higher DPEP1 expression (Fig. 1i). Consistently, Gene Set Enrichment Analysis (GSEA) suggested that high expression level of DPEP1 was correlated with poor prognosis (Fig. 1j). Univariate and multivariate Cox regression analysis revealed that vascular invasion, distant metastasis, recurrence, and high DPEP1 expression were independent risk factors for survival in children with HB (Fig. 1k and Table 2). Taken together, these findings suggest that DPEP1 might play a vital function in the tumorigenesis of HB.

**Table 1:** Association of DPEP1, miR-193a-5p expression, and clinicopathological features

Clinicopathological features	No. of cases	DPEP1		*P* value	MiR-193a-5p		*P* value
		Low (*n* = 30)	High (*n* = 40)		Low (*n* = 39)	High (*n* = 31)	
Age (years)							
≤Median	35	13	22	0.334	21	14	0.4704
>Median	35	17	18		18	17	
Gender							
Male	44	18	26	0.668	23	21	0.4508
Female	26	12	14		16	10	
Tumor size							
≤10 cm	36	16	20	0.782	19	17	0.6108
>10 cm	34	14	20		20	14	
Vascular invasion							
Absent	55	28	27	**0.009**	25	30	**0.0327**
Present	15	2	13		14	1	
AFP							
≤100 ng/ml	36	16	20	0.782	23	13	0.1565
>100 ng/ml	34	14	20		16	18	
Histologic type							
Epitheliated	52	22	30	0.875	29	23	0.9874
Mixed	18	8	10		10	8	
Metastasis							
Absent	52	27	25	**0.009**	23	29	**0.001**
Present	18	3	15		16	2	
Recurrence							
Absent	57	29	28	**0.006**	28	29	**0.0201**
Present	13	1	12		11	2	
SIOPEL + GPOH							
Standard-risk	40	22	18	**0.018**	14	26	**0.000**
High-risk	30	8	22		25	5	

Bold values indicates statistical significance, *P* < 0.05

**Table 2:** Univariate and multivariate analyses of overall survival of hepatoblastoma

Clinicopathological features	Univariate analyses		*P* value	Multivariate analyses		*P* value
	HR	95% CI		HR	95% CI	
Age (years)						
<Median	1.027	0.436–2.420	0.951			
>Median						
Gender						
Male	0.882	0.365–2.130	0.781			
Female						
Tumor size						
≤10 cm	1.674	0.693–4.044	0.252			
>10 cm						
AFP						
≤100 ng/ml	0.972	0.225–4.192	0.969			
>100 ng/ml						
Histologic type						
Epitheliated type	0.868	0.317–2.378	0.783			
Mixed						
Vascular invasion						
Absent	2.863	1.136–7.217	**0.026**	1.000		
Present				3.914	1.390–11.022	**0.010**
Distant metastasis						
Absent	7.841	3.133–19.623	**0.000**	1.000		
Present				6.923	2.135–18.546	**0.001**
Recurrence						
Absent	7.171	2.989–17.204	**0.000**	1.000		
Present				4.158	1.481–11.676	**0.007**
SIOPEL + GPOH						
Standard-risk	7.143	2.396–21.292	**0.000**	1.000		
High-risk				0.820	0.158–4.250	0.814
DPEP1 expression						
Low	3.565	1.197–10.611	**0.022**	1.000		
High				6.162	1.952–19.727	**0.002**

Bold values indicates statistical significance, *P* < 0.05

### Knockdown of DPEP1 suppresses HB cell proliferation, migration, and invasion in vitro

**Fig. 2:**
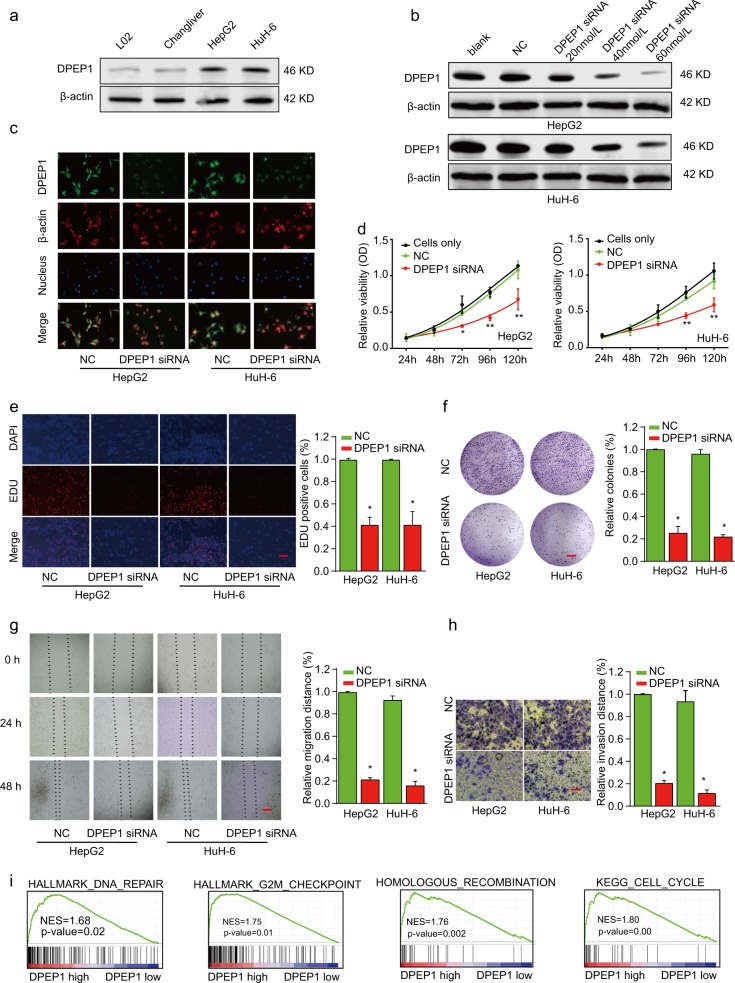
Knockdown of DPEP1 suppresses HB cell proliferation, migration, and invasion. **a** Western blot analysis of DPEP1 expression in normal liver cell lines (L02 and Chang Liver) and HB cell lines (HepG2 and HuH-6). **b** Western blot analysis of DPEP1 expression in HepG2 or HuH-6 cells transfected with negative control (NC), or different concentration of DPEP1 siRNA. **c** Confirmation of DPEP1 knockdown in HB cell lines by immunofluorescence. **d** Cell proliferation of HepG2 or HuH-6 cells transfected with NC or DPEP1 siRNA was analyzed by CCK-8 assay. **e** Cell proliferation of HepG2 or Huh-6 cells transfected with NC or DPEP1 siRNA was analyzed by EDU staining assay. Scale bars, 50 μm. **f** Colony formation of HepG2 or HuH-6 cells transfected with NC or DPEP1 siRNA. Scale bars, 8 mm. **g** The migration capability of HepG2 or HuH-6 cells transfected with NC or DPEP1 siRNA was analyzed by wound-healing assay at indicated time points. Scale bars, 500 μm. **h** The invasion capability of HepG2 or HuH-6 cells transfected with NC or DPEP1 siRNA was analyzed by transwell assay. Scale bars, 50 μm. **i** The gene set enrichment analysis (GSEA) disclosed several cell cycle enrichment pathways between DPEP1 high group and low group. *P < 0.05, **p < 0.01

To investigate the biological function of DPEP1 in HB cells, we examined the DPEP1 expression in normal liver cells (L02 and Chang Liver) and HB cell lines (HepG2 and HuH-6). Western blot result showed that the expression levels of DPEP1 were much higher in HepG2 and HuH-6 cells than that in normal liver cells (Fig. 2a). Next, we employed small interfering RNA (siRNA) to knockdown DPEP1 expression in HepG2 and HuH-6 cells (Fig. 2b and Supplementary Fig. S1). Then, DPEP1 immunofluorescence analysis was performed. DPEP1 protein expression was mainly localized in the cytoplasm of HB cells. The results of the DPEP1 immunofluorescence assay further confirmed the significantly downregulated expression of DPEP1 after transfection with DPEP1 siRNA (Fig. 2c). Cell Counting Kit-8 (CCK-8) proliferation assay (Fig. 2d), 5-ethynyl-2′-deoxyuridine (EDU) staining (Fig. 2e), and colony formation assays (Fig. 2f) demonstrated that cell proliferation ability was dramatically suppressed after silencing DPEP1. In addition, the migratory and invasive ability were also remarkably decreased in HepG2 or HuH-6 cells transfected with DPEP1 siRNA, as demonstrated by wound-healing (Fig. 2g) and transwell assays (Fig. 2h). GSEA and Kyoto Encyclopedia of Genes and Genomes (KEGG) analysis indicated that the levels of DPEP1 expression were positively correlated with DNA repair, G2M checkpoint, recombination, or cell cycle (Fig. 2i). On the contrary, we demonstrated that upregulation of DPEP1 promoted HB cell proliferation, migration, and invasion in vitro (Supplementary Fig. S2). Overall, we concluded that DPEP1 could promote HB cell proliferation, migration, and invasion.

### Knockdown of DPEP1 inhibits tumor formation in vivo

**Fig. 3:**
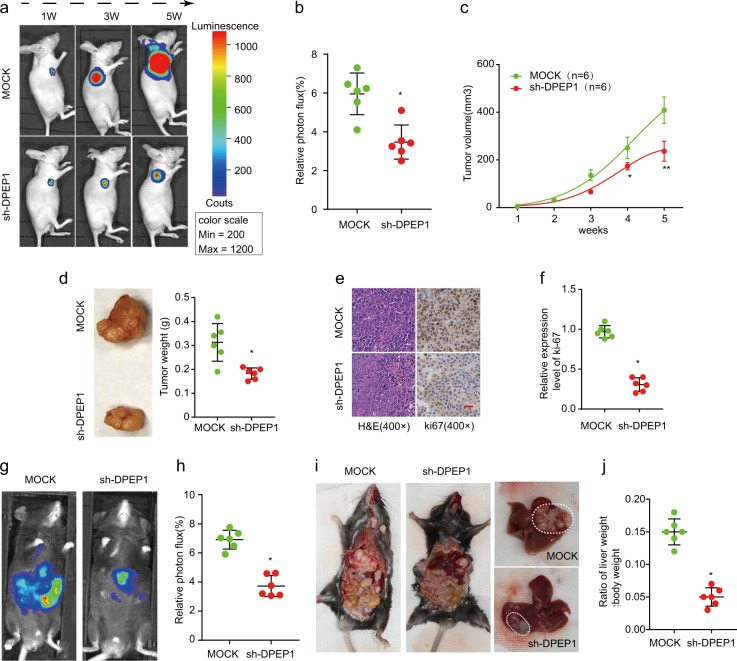
Knockdown of DPEP1 inhibits tumor formation in vivo. **a** Representative image of Luciferase signal emission from mice implanted with HepG2 cells stably knockdown DPEP1 or mock control at indicated time points. **b** Relative photon flux in mock and sh-DPEP1 groups were quantified and analyzed using the IVIS imaging system 5 weeks after implantation. **c** The growth curves of tumor were determined based on the tumor size measured every week. **d** Representative photographs of HB tumors and tumor weights in mock and sh-DPEP1 groups were analyzed at week 5. **e**, **f** Representative immunohistochemical staining images and relative expression levels of Ki-67 in tumors from mock or sh-DPEP1 group. Scale bars, 200 μm. **g**, **h** Relative photon flux of orthotopic transplanted tumor in mock and sh-DPEP1 groups were quantified and analyzed using the IVIS imaging system 5 weeks after implantation. **i** Representative images of orthotopic tumors in the liver after dissection. **j** The ratio of liver weight/body weight in mice orthotopically implanted Hepa1-6 cells stably knockdown DPEP1 or mock control was analyzed 5 weeks after implantation. *P  < 0.05, **p < 0.01

To verify the effect of DPEP1 downregulation on HB tumorigenesis in vivo, HepG2 cells with stable DPEP1 knockdown (transfected with DPEP1 shRNA (short hairpin RNA)) or mock control (transfected with MOCK-shRNA) were implanted into nude mice. Knockdown of DPEP1 significantly inhibited tumor growth as shown by luciferase photon flux and tumor volumes at different time points after implantation (Fig. 3a–c). Consistently, the tumors in sh-DPEP1 group had markedly lower tumor weight compared with those in mock control group (Fig. 3d). We further examined the proliferation marker Ki-67 and found its expression was also decreased in the sh-DPEP1 group by IHC analysis (Fig. 3e, f).

To further confirm the above results in a system that more accurately mimic the in vivo environment, an orthotopic HB transplantation model in C57BL/6 mice was established using subcutaneous tumor of nude mice. Fluorescence images demonstrated that DPEP1 knockdown resulted in significantly decreased tumor size (Fig. 3g, h). The orthotopic xenograft tumors in sh-DPEP1 group had less tumor metastasis and lower liver weight/body weight ratios (Fig. 3i, j). These results further indicate the oncogenic ability of DPEP1 in HB.

### DPEP1 activates PI3K/Akt/mTOR signaling in HB

**Fig. 4:**
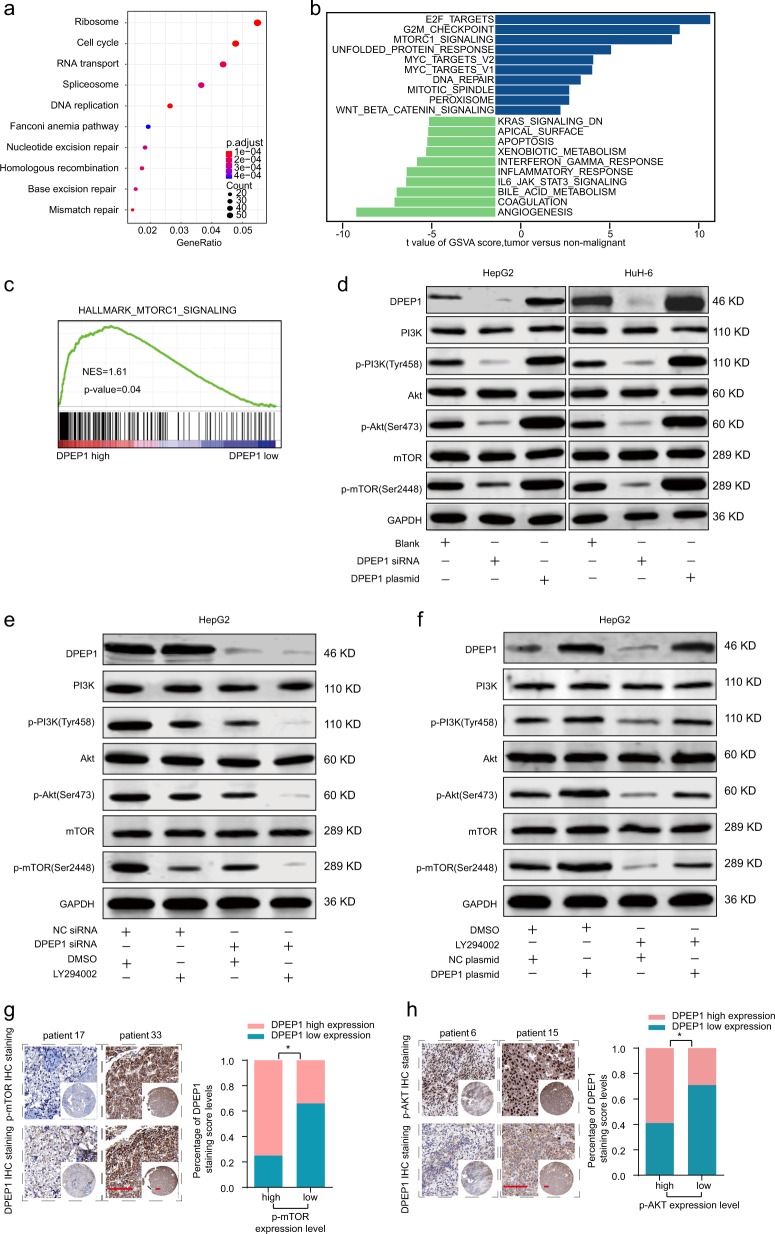
Functional and pathway enrichment analysis of DPEP1 and DPEP1 activates PI3K/Akt/mTOR signaling in HB. **a** KEGG enrichment analysis disclosed several enrichment pathways in HB cohort. **b** Gene set variation analysis (GSVA) comparison of signaling pathways with differentially expressed genes between DPEP1 high expression and low expression groups. **c** GSEA analysis the enrichment of mTORC1 signaling DPEP1 high expression and low expression groups. **d** Expression levels of PI3K, p-PI3K (Tyr458), Akt, p-Akt (Ser473), mTOR, and p-mTOR (Ser2448) in HepG2 and HuH-6 transfected with DPEP1, DPEP1 siRNA, or cells only (Blank) were analyzed by western blot. **e** Expression levels of PI3K, p-PI3K (Tyr458), Akt, p-Akt (Ser473), mTOR, and p-mTOR (Ser2448) in HepG2 transfected with DPEP1 siRNA, NC siRNA, LY294002, or DMSO were analyzed by western blot. **f** Expression levels of PI3K, p-PI3K (Tyr458), Akt, p-Akt (Ser473), mTOR, and p-mTOR (Ser2448) in HepG2 transfected with DPEP1 plasmid, NC plasmid, LY294002, or DMSO were analyzed by western blot. The representative result of at least three independent experiments was shown. **g** Representative IHC staining of DPEP1 and mTOR in HB tissues from ZZU cohort. Scale bars, 50 μm. *P < 0.05. **h** Representative IHC staining of DPEP1 and Akt in HB tissues from ZZU cohort. Scale bars, 50 μm. *P < 0.05

To further study the potential function mechanism of DPEP1 in HB, bioinformatics analysis was performed based on GEO HB cohort. Heatmap of different gene expression analysis was shown in Supplementary Fig. S3a. KEGG enrichment analysis disclosed several enriched signaling pathways (Fig. 4a). Gene set variation analysis (GSVA) and GSEA indicated that the mTORC1 signaling pathway served as one of the major enriched pathways with high DPEP1 expression (Fig. 4b, c). We found that the protein expression levels of phosphorylated (p)-PI3K, p-AKT, and p-mTOR were significantly decreased in DPEP1-knockdown group and increased in DPEP1 overexpression group, respectively (Fig. 4d). Furthermore, LY294002 (a PI3K signaling inhibitor) was utilized to inhibit the PI3K/Akt/mTOR pathway and the results demonstrated that DPEP1 downregulation could further enhance the inhibitory effect of LY294002, while DPEP1 overexpression could reverse the inhibitory effect of LY294002 in HepG2 (Fig. 4e, f). Similar results were observed in HuH-6 (Supplementary Fig. S3b, c). These results corresponded to the proliferation and invasion abilities of HepG2 cells (Supplementary Fig. S3d). IHC staining of DPEP1 was inversely associated with the IHC scores of mTOR and Akt in HB tissues (Fig. 4g, h).To make our conclusion more solid and rigorous, insulin-like growth factor 1 (IGF-1) was utilized to agonist the PI3K/Akt/mTOR pathway and the results demonstrated that DPEP1 downregulation could reverse the agonist effect of IGF-1 in HepG2 (Supplementary Fig. S4a). Furthermore, rescue experiments in vitro found that activating PI3K/Akt/mTOR pathway using IGF-1 reverted the suppressive effects of DPEP1 silencing on the proliferation and invasion abilities of HepG2 cells (Supplementary Fig. S4b). In addition, we also examined whether DPEP1 regulated the Wnt/β-catenin signaling pathway. GSEA analysis and western blot showed that there was no significant correlation between the expression of Wnt/β-catenin signaling protein and DPEP1 expression (Supplementary Fig. S5). Overall, our results demonstrate that DPEP1 exerts its function via activating PI3K/Akt/mTOR signaling pathway in HB.

### DPEP1 is a direct target of miR-193a-5p in HB

**Fig. 5:**
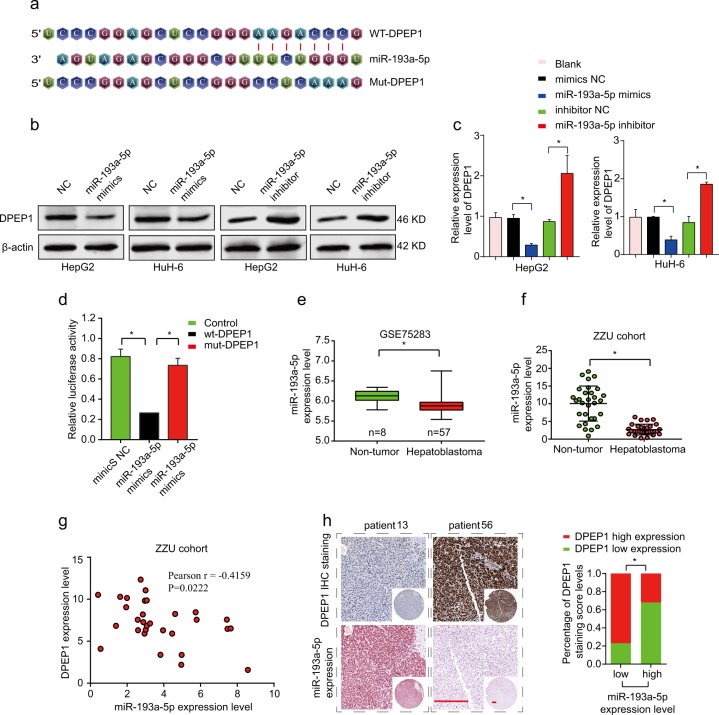
DPEP1 is a direct target of miR-193a-5p. **a** Diagram of the potential binding sequences of miR-193a-5p on the 3′-UTR of DPEP1 and the mutated sequences of DPEP1 3′-UTR. **b**, **c** Western blot (**b**) and qRT-PCR analysis (**c**) the protein and mRNA expression levels of DPEP1 in HepG2 or HuH-6 cells transfected with miR-193a-5p mimics, miR-193a-5p inhibitor, or negative control (NC). **d** WT 3′-UTR or mutated 3′-UTR of DPEP1 was constructed into luciferase reporter vector and co-transfected with miR-193a-5p mimics into HEK293 cells. Relative luciferase activity was assessed 48 h after transfection. **e**, **f** The expression levels of miR-193a-5p in GEO database GSE75283 and ZZU cohorts. **g** Pearson’s correlation analysis of the relationship between DPEP1 expression level and miR-193a-5p expression level in ZZU cohort. **h** Representative IHC staining of DPEP1 and miR-193a-5p in HB tissues from ZZU cohort (left panel) and quantification of DPEP1 staining scores in HB patients from ZZU cohort with high or low miR-193a-5p expression. Scale bars, 50 μm. *P < 0.05

To explore the target miRNAs of DPEP1 in HB, we searched for potential target miRNAs by bioinformatics with publicly available databases: miRBase (http://www.mirbase.org/) and TargetScan (http://www.targetscan.org/). Bioinformatics analysis demonstrated that miR-193a-5p had a complementary binding sites targeting 3′-UTR of DPEP1 (Fig. 5a). Western blot and quantitative real-time PCR (qRT-PCR) results indicated the expression levels of miR-139a-5p were negatively correlated with DPEP1 expression in HepG2 and HuH-6 cells. While overexpression miR-193a-5p inhibited DPEP1 expression, inhibition of miR-193a-5p significantly enhanced DPEP1 expression (Fig. 5b, c). Further, the relative luciferase activity in HEK293 cells transfected with the reporter vector containing wild-type (WT) 3′-UTR of DPEP1 was markedly reduced compared to that in HEK293 cells transfected with reporter vector containing mutated 3′-UTR of DPEP1, indicating the direct interaction between miR-193a-5p and 3′-UTR of DPEP1 mRNA (Fig. 5d). Moreover, we confirmed that miR-193a-5p was markedly low expressed in tumor tissues through HB miRNA microarray analysis (GSE75283), which was further validated in our own fresh HB tissue specimens through qRT-PCR analysis (Fig. 5e, f). In addition, the Pearson’s correlation coefficient analysis showed that there was a remarkably negative correlation between miR-193a-5p and DPEP1 expression (Fig. 5g). In situ hybridization (ISH) staining of miR-193a-5p was inversely associated with the IHC scores of DPEP1 in HB tissues (Fig. 5h). In summary, miR-193a-5p directly targets DPEP1 and negatively regulates its expression.

### Overexpression of miR-193a-5p suppresses HB cell proliferation and invasion through targeting DPEP1

**Fig. 6:**
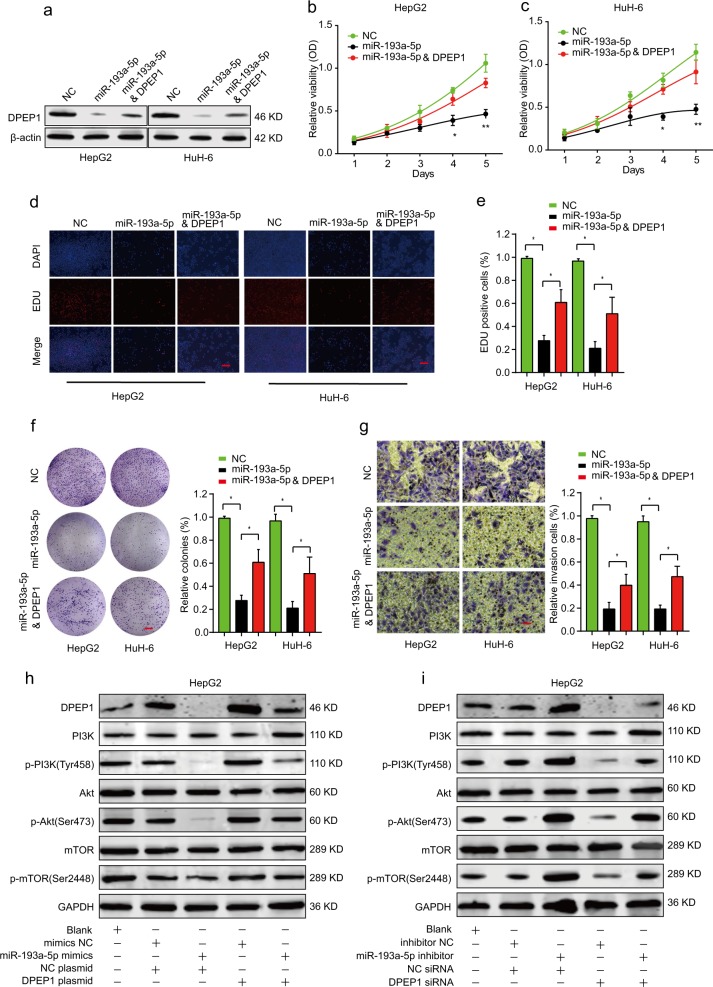
Overexpression of miR-193a-5p suppresses HB cell proliferation and invasion through targeting DPEP1. HepG2 or HuH-6 cells were transfected with negative control (NC), miR-193a-5p mimics, or miR-193a-5p mimics and DPEP1 overexpression plasmid. **a** The protein levels of DPEP1 in different groups were analyzed by western blot. **b-e** Cell proliferation of HepG2 or HuH-6 cells was assessed by CCK-8 assay (**b**, **c**) or EDU staining assay. Scale bars, 50 μm. **d**–**f** Cell colony capacity of HepG2 or HuH-6 in different groups was analyzed by colony formation assay. Scale bars, 8 mm. **g** Cell invasion ability of HepG2 or HuH-6 cells in different groups was analyzed by transwell assay. Scale bars, 50 μm. **h** Expression levels of PI3K/p-PI3K (Tyr458), Akt/p-Akt (Ser473), and mTOR/p-mTOR (Ser2448) in HepG2 transfected with negative control mimics, miR-193a-5p mimics, negative control plasmid, or DPEP1 plasmid were analyzed by western blot. **i** Expression levels of PI3K/p-PI3K (Tyr458), Akt/p-Akt (Ser473), and mTOR/p-mTOR (Ser2448) in HepG2 transfected with negative control inhibitor, miR-193a-5p inhibitor, negative control siRNA, or DPEP1 siRNA were analyzed by western blot. The representative result of at least three independent experiments was shown. *P < 0.05, **p < 0.01

To demonstrate the effects of miR-193a-5p/DPEP1 axis on HB cells, we transfected HepG2 or HuH-6 cells with miRNA negative control (NC), miR-193a-5p mimics, or miR-193a-5p mimics and DPEP1 plasmid. Western blot exhibited that while miR-193a-5p significantly inhibited DPEP1 expression, DPEP1 overexpression partially rescued DPEP1 expression in miR-193a-5p mimics and DPEP1 plasmid group (Fig. 6a). CCK-8, EDU staining, and colony formation experiments demonstrated that miR-193a-5p overexpression suppressed proliferative potential in both HepG2 and HuH-6 cells, but this suppression effect could be partially reversed by overexpressing DPEP1 (Fig. 6b–f). Meanwhile, DPEP1 overexpression partially rescued the cell invasion of HepG2 or HuH-6 cells with miR-193a-5p overexpression (Fig. 6g).

We further evaluated the function of miR-193a-5p/DPEP1 axis on PI3K/Akt/mTOR signaling. The results suggested that miR-193a-5p mimics inhibited the activation of the PI3K/Akt/mTOR signaling and DPEP1 overexpression could reverse the inhibitory effect (Fig. 6h, i). Taken together, these findings demonstrate that miR-193a-5p suppresses HB cell proliferation and invasion in vitro through targeting DPEP1, which involves the PI3K/Akt/mTOR signaling pathway.

### Low miR-193a-5p expression is associated with poor prognosis in HB patients

**Fig. 7:**
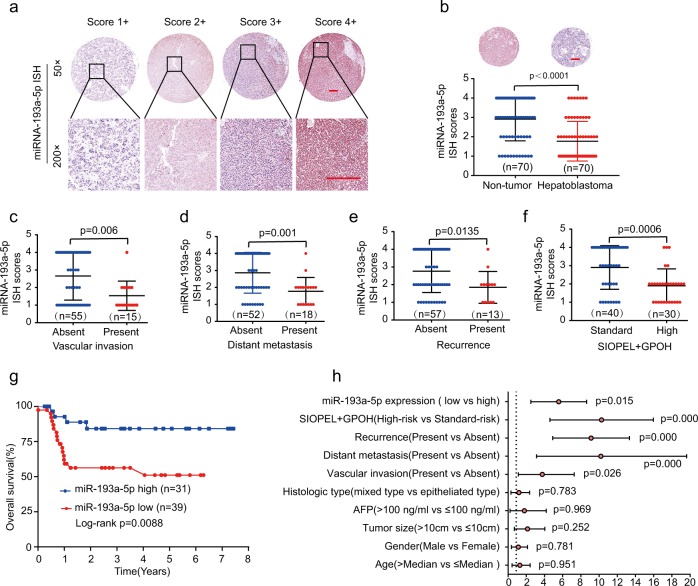
Low miR-193a-5p expression is associated with poor prognosis in HB patients. **a** Representative MiR-193a-5p staining patterns with different staining scores in HB TMA. Scale bars, 100 μm. **b** The miR-193a-5p expression levels in HB tissues or non-tumor normal tissues based on ISH scores. Scale bars, 200 μm. **c–f** The comparison of miR-193a-5p ISH staining score distributions between HB patients with absent or present of vascular invasion (**c**), distant metastasis (**d**), recurrence (**e**), or high or low of SIOPEL + GPOH risk stratification (**f**). **g** Kaplan–Meier survival analysis of OS in HB patients with high or low levels of miR-193a-5p. **h** Univariate Cox analysis of the relationship between clinicopathological features and miR-193a-5p expression

We analyzed miR-193a-5p expression in an HB TMA cohort by ISH. According to the different staining intensities, we scored the staining intensity of miR-193a-5p with four different levels (1+ to 4+), as shown in Fig. 7a. The results showed that miR-193a-5p was dramatically downregulated in HB tissues in comparison with that in non-tumor tissues (Fig. 7b). In addition, miR-193a-5p expression was associated with vascular invasion, distant metastasis, recurrence, and SIOPEL+ GPOH risk stratification (Fig. 7c–f, Table 1). Furthermore, Kaplan–Meier analysis concluded that low levels of miR-193a-5p related to poor OS rates in HB patients (Fig. 7g). Meanwhile, univariate Cox analysis of OS demonstrated that vascular invasion, distant metastasis, recurrence, SIOPEL + GPOH risk stratification, and miR-193a-5p were important prognostic factors (Fig. 7h). In summary, these results suggest that miR-193-5p is critical for the prognosis of HB.

## Discussion

Emerging evidences have shown that DPEP1 is highly expressed and plays vital roles in the progression of multiple tumors. For instance, previous study has reported that the high expression level of DPEP1 was associated with poor prognosis of colorectal cancer^7^, and Park et al.^8^ reported that DPEP1 enhanced colon cancer metastasis by controlling E-cadherin plasticity. Consistent with these results, our data indicated that DPEP1 had an ectopic overexpression in HB tissues and was associated with poor prognosis in HB afflicted children (Fig. 1). In addition, functional assays revealed that DPEP1 silencing could significantly suppress the HB cell proliferative, colony formation, migration, and invasion capacity in vitro, and knockdown of DPEP1 in vivo resulted in decreased HB tumor growth (Figs. 2 and 3). In contrast, DPEP1 expression appears downregulated in pancreatic ductal adenocarcinoma, and the overexpression of DPEP1 inhibited tumor cell invasiveness and acted as a potential tumor suppressor^20^. Toiyama et al.^6^ reported that overexpression of DPEP1 inhibited cancer cell invasiveness in the early stages of colon carcinogenesis^6^. The above-mentioned reports suggest that DPEP1 may exert an oncogene or tumor suppressor function depending on the circumstances and tissue-specific expression pattern.

Subsequently, we verified DPEP1 was targeted by miR-193a-5p (Fig. 5). Previous researches have elucidated that miR-193a-5p played a tumor suppression role in many cancer types. For example, miR-193a-5p overexpression could suppress breast cancer proliferation and metastasis^21^. MiR-193a-5p could suppress tumor proliferation and enhance radio-sensitivity in esophageal squamous cell carcinoma^22^. Yu et al.^23^ concluded that miR-193a-5p could inhibit lung cancer metastasis. Additionally, previous publications had documented that miR-193a-5p was related to chemotherapy drug resistance^18^. Consistent with these findings, our data indicated that miR-193a-5p was expressed at low levels and was significantly associated with poor clinical outcomes of HB-afflicted children (Fig. 7). In addition, cell proliferative and invasive capacities were inhibited by miR-193a-5p overexpression, which could be reversed by the overexpression of DPEP1 (Fig. 6). Thus, these findings uncovered that miR-193a-5p had a low expression in HB, and miR-193a-5p overexpression could inhibit progression of HB by targeting DPEP1.

**Fig. 8:**
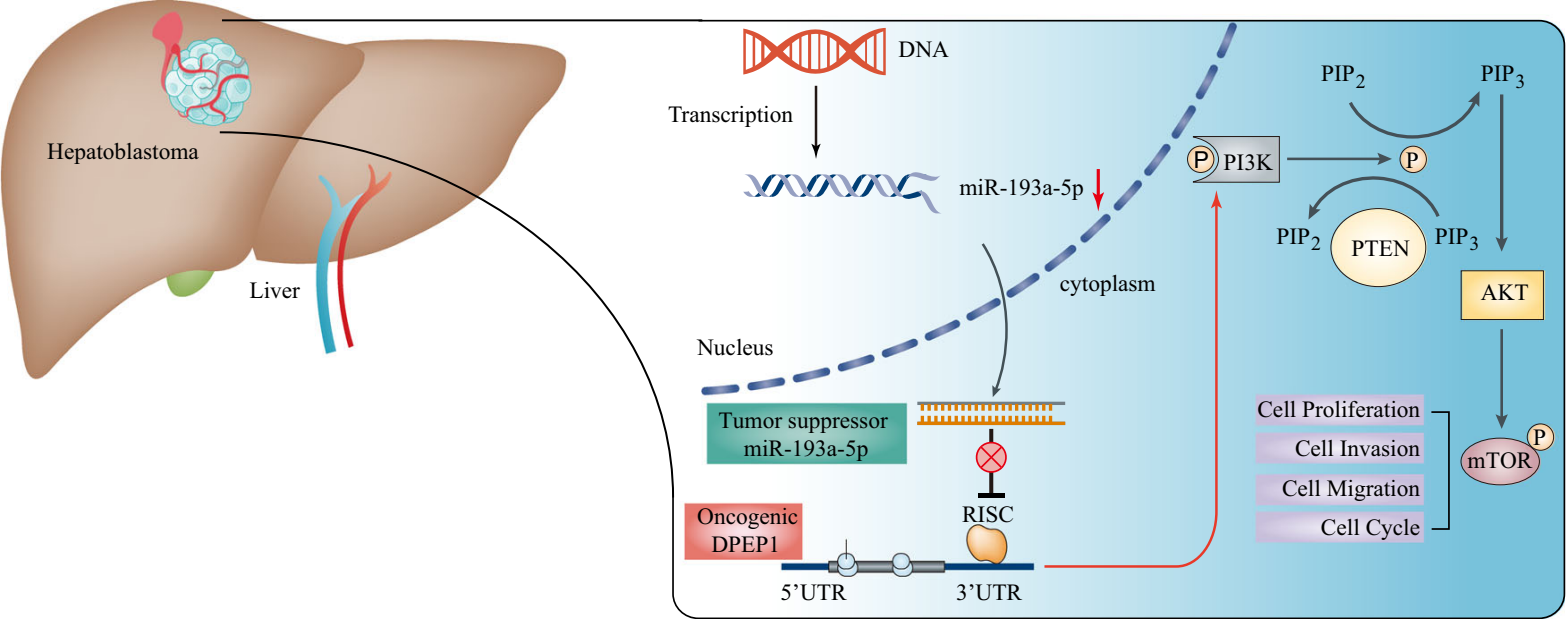
A mechanism diagram depicting that the miR-193a-5p/DPEP1 axis affects the progression of HB through the PI3K/Akt/mTOR signaling

Bioinformatics analysis and experimental verification confirmed that DPEP1 acted as an oncogene in HB by activating PI3K/Akt/mTOR signaling pathways (Fig. 4). PI3K/Akt/mTOR signaling is widely considered as a central adjustment factor of cell proliferation, survival, and metabolism^24^. A great number of reports have shown that PI3K/Akt/mTOR signaling plays a pivotal role in tumorigenesis, including HB^25,26^. For instance, tamoxifen down-regulating survivin expression in HB cell line HepG2 was mediated by PI3K/Akt/mTOR to induce apoptosis^27^. Li et al.^28^ claimed that downregulation of the PI3K/Akt/mTOR signaling could induce apoptosis in HB cancer HepG2 cells. Zhang et al.^29^ also showed that PP7 could inhibit PI3K/AKT/mTOR signaling and induce autophagic cell death in HepG2. In our study, overexpression of DPEP1 was positively correlated with the expression levels of PI3K/Akt/mTOR signaling-associated molecules (Fig. 4d–f), while miRNA-193a-5p mimics suppressed the activation of the PI3K/AKT/mTOR signaling. Moreover, DPEP1 overexpression could reverse the inhibitory effect of miRNA-193a-5p (Fig. 6h, i). Overall, our results provide solid evidences that miR-193a-5p/DPEP1 is involved in HB progression through controlling the PI3K/Akt/mTOR signaling (Fig. 8).

In summary, we report that miR-193a-5p could regulate DPEP1 expression in tumorigenicity and progression of HB via PI3K/Akt/mTOR signaling. The miR-193a-5p/DPEP1 axis may be used as an effective therapeutic and prognostic biomarker for HB-afflicted patients.

## Materials and methods

### Patients and specimens

The HB tissues and adjacent normal tissues were collected from HB patients (*n* = 70) recruited into the First Affiliated Hospital of Zhengzhou University between April 2011 and December 2018. TMAs containing 70-paired paraffin-embedded HB specimens (ZZU cohort) were prepared using 1.5-mm-diameter needle puncture samples. The follow-up and clinicopathological data were listed in Table 1. The study was approved by the Ethics Review Committee of the First Affiliated Hospital of Zhengzhou University. All patients were informed and educated of the procedure and provided written consent.

### Cell culture

The HB cell lines (HepG2), hepatocellular carcinoma cell lines (Hepa1-6), and embryonic kidney cell lines (HEK293) were acquired from Cell Bank, Shanghai Institute of Cell Biology (Shanghai, China). Human HB cell line Huh-6, normal liver cell line L02, and Chang Liver were purchased from FuHeng Cell Center (Shanghai, China). Cells were maintained in Dulbecco’s modified Eagle’s medium (DMEM) medium supplemented with 10% fetal bovine serum (FBS) and 100 U/ml penicillin/streptomycin (Corning, NY, USA) in a humid incubator with 5% carbon dioxide at 37 °C. Cells were maintained <6 months before the experiments were conducted. The details of these cells were shown in Supplementary Table 1.

### Dataset acquisition and process

TCGA database (http://gdc-portal.nci.nih.gov/) and GTEX database (https://gtexportal.org/) were used to explore the expression of DPEP1 in pan-cancers. Two sets of microarrays (GSE75271, GSE75283) were extracted from the GEO database (http://www.ncbi.nlm.nih.gov/geo/) to analyse the expression of DPEP1 and miR-193a-5p in HB cancer tissues and normal tissues (Supplementary Table 2).

### IHC staining and in situ hybridization

IHC was carried out as described previously^30^. Based on the proportion of positive cells and the different staining intensity, we established a semi-quantitatively scoring system, the proportion of positive cells were scored as follows: 0—none, 1+—<25%, 2+—25–50%, 3+—50–75%, and 4+—75–100%. The staining intensity was scored as follows: 0—none, 1+— weak, 2+—medium, and 3+—strong. The total score was calculated by multiplying the two sub-scores, and samples with scores of 0–6 were deemed as low expression and 7–12 scores were classified as high expression. Blind quantification of DPEP1 staining was performed by two independent pathologists.

ISH was conducted using digoxygenin-labeled miR-193a-5p ISH probes obtained from Boster (Wuhan, China). miR-193a-5p expression in HB TMA was detected based on fluorescence. The different intensities and proportion of miR-193a-5p dyeing were divided into four scores: 1+ to 2+ scores were defined as low expression, while 3+ to 4+ scores represented high expression. Supplementary Table 3 lists the information of the antibodies utilized used in this study.

### Western blot

RIPA buffer was utilized to extracted total protein from HB cells. Following extraction, BCA assays (Beyotime, Shanghai, China) were performed to quantify all proteins. Twenty micrograms of protein samples were loaded onto 12% sodium dodecyl sulfate-polyacrylamide gel electrophoresis. Following separation, the samples were transferred from the gel to the nitrocellulose membranes (Millipore, MA, USA) and blocked with 5% bovine serum albumin/PBST for 1 h. The membranes were then incubated with anti-β-actin, DPEP1, or indicated antibodies at 4 °C overnight. After washed with PBST, the secondary antibody incubation was performed for 2 h and the membranes were exposed with the photographic film for visualization. Supplementary Table 3 listed the information of antibodies.

### Oligonucleotide and plasmid transfection

DPEP1 siRNAs (si-DPEP1), DPEP1 plasmid (DPEP1), miR-193a-5p mimics, miR-193a-5p inhibitor, and the corresponding NCs were acquired from GenePharma (Shanghai, China). The sequence of these siRNAs was listed in Supplementary Table 4. Cells were transfected using Lipofectamine 2000 (Thermo Fisher, CA, USA) following the manufacturer’s protocols. Western blotting and qPCR were performed at 48–72 h to determine the transfection efficiency.

### Quantitative real-time PCR

Total RNA was extracted using Trizol (Life Technologies, CA, USA). TransScript First-Strand cDNA Synthesis SuperMix (TransGen, Beijing, China) was used to generate complementary DNA (cDNA). qRT-PCR reactions were conducted using PowerUp SYBR Green Kit (ABI, Foster City, USA) and QuantStudio 6 System (ABI, Foster City, USA). The relative gene expression was normalized to control using 2^−ΔΔCt^ method.

### Cell proliferation and colony formation assays

Cell proliferation was assessed using CCK-8 Kit (Beyotime, China). The DNA synthesis rate was evaluated through Edu staining assay (Ribobio, Guangzhou, China). Cell colony formation ability after DPEP1 silencing was assessed by colony formation assay. Cells transfected with si-DPEP1, miR-193a-5p, miR-193a-5p and DPEP1 plasmid, or NC controls were seeded in 6-well plates and incubated for 2 weeks. Then, cells were fixed with formaldehyde and stained with 0.1% crystal violet. The colonies with more than 50 cells were counted by light microscopy.

### Cell migration and invasion assays

Wound-healing assay was conducted to determine the migration ability. HepG2 and HuH-6 cells (5 × 10^6^) were seeded into six-well plates. A 1 mm wide wound was created using a 200-μL sterile tip after 90% confluence was reached. The wounded areas were observed and photographed every 24 h under microscope.

Cell invasion assay was conducted utilizing transwell chambers coated with Matrigel (BD, NJ, USA). Upper chambers were seeded at 1 × 10^4^ transfected cells using serum-free media, and the lower chamber was filled with DMEM with 10% FBS. After 24 h, the invasive cells were fixed, stained, photographed, and quantified.

### Luciferase assays

Both WT and mutant 3′-UTR of the DPEP1 mRNA were cloned into the luciferase reporter vector (psiCHECK-2, Promega, WI, USA). HEK293 cells were co-transfected with miR-193a-5p mimics and either DPEP1-wt or DPEP1-mut vectors. After 48 h, the relative luciferase activity was analyzed using the Dual-Luciferase Reporter Assay System (Promega, WI, USA).

### Lentiviral transduction and vector construction

The CDS of human DPEP1 were cloned into pcDNA3.1 (+) to construct DPEP1 overexpression vectors. HepG2 and Hepa1-6 cells were infected with lentivirus containing DPEP1 shRNA or control lentivirus for 4 days and then screened for stably knockdown cells using puromycin (Santa Cruz Biotechnology, Dallas, TX, USA).

### Tumor xenografts

Mice experiments were approved by the Animal Health Committee of Zhengzhou University. The nude mice (male, 4–6 weeks old) and C57BL/6 mice (male, 8 weeks old) were purchased from Beijing Vital River Laboratory (Beijing, China). Cells transfected with DPEP1 knockdown (sh-DPEP1) and empty lentivirus control (MOCK) were subcutaneously implanted into the lower flank of nude mice. HepG2 cell lines were selected to establish xenograft model in nude mice. Liver orthotopic transplantation model was established with Hepa1-6 cells. After 1–2 weeks, a mouse model of orthotopic transplantation of the liver was established by removing the subcutaneous tumor derived from Hepa1-6 cells and transplanting 1 mm^3^ samples into the liver left lobe of the C57BL/6 mouse (8 weeks old). Tumor growth was examined every week. Mice were euthanized at 5 weeks post orthotopic transplantation. Photographs were taken using the IVIS Lumina II system. The tumor tissues were weighed and extracted for further IHC staining.

### Statistical analysis

All statistical analyses were conducted using SPSS (Version 23.0, IBM, WA, USA). Differences between two groups were analyzed by Student’s *t* test. Clinicopathological characteristics in HB were analyzed by *χ*^2^ tests. OS was calculated with Kaplan–Meier curves and log-rank tests. Univariate and multivariate Cox regression analyses were performed to identify the independent prognostic factors. Correlation was performed by Spearman’s rank analysis with GraphPad Prism 7.0 (San Diego, CA, USA). A *p* value <0.05 was statistically significant.

## Supplementary information

supplementary tables 1-4.

Supplementary Figure legends.

Supplementary Figure S1.

Supplementary Figure S2.

Supplementary Figure S3.

Supplementary Figure S4.

Supplementary Figure S5.

hepg2-STR Profiling

CHANG LIVER-STR Profiling

HUH-6-STR Profiling

L02-STR Profiling
