# Supplementary material for: DPEP1 is a direct target of miR-193a-5p and promotes hepatoblastoma progression by PI3K/Akt/mTOR pathway
Source: Cell Death Dis. 2019 Sep 20;10(10):701. doi: 10.1038/s41419-019-1943-0 (PMC6754441; doi:10.1038/s41419-019-1943-0)
Supplement: Supplementary file 1 — supplementary tables 1-4. [file 41419_2019_1943_MOESM1_ESM.docx]

**Supplementary Table 1. Cell lines used in this study**

| **Cell lines** | **Cell type** | **Source** | **Country** |
| --- | --- | --- | --- |
| HepG2 | Hepatoblastoma cell | Cell Bank of the Chinese  Academy of Science | China |
| HuH-6 | Hepatoblastoma cell | Fuheng cell center | China |
| Chang liver | Normal liver cell | Fuheng cell center | USA |
| L02 | Normal liver cell | Fuheng cell center | China |
| HEK293 | Embryonic kidney cell | Cell Bank of the Chinese  Academy of Science | China |
| Hepa1-6 | Hepatocellular carcinoma cell | Cell Bank of the Chinese  Academy of Science | China |

**Supplementary Table 2. GEO information used in this study**

| **GEO ID** | **Platforms** | **Non-tumor** | **Tumor** | **Year** | **Country** |
| --- | --- | --- | --- | --- | --- |
| GSE75271 | Affymetrix mRNA microarray | 5 | 50 | 2016 | USA |
| GSE75283 | Agilent miRNA microarray | 8 | 57 | 2017 | USA |
| Total |  | **13** | **107** |  |  |

**Supplementary Table 3. Information on antibodies used in this study**

| **Antibody** | **WB** | **IHC** | **Specificity** | **Company** |
| --- | --- | --- | --- | --- |
| β-actin | 1:5000 | / | Mouse monoclonal | Proteintech Group, China |
| DPEP1 | 1:2000 | 1:100 | Rabbit Polyclonal | Proteintech Group, China |
| Ki-67 | / | 1:500 | Rabbit Polyclonal | Proteintech Group, China |
| PI3K | 1:1000 | / | Rabbit Polyclonal | Proteintech Group Chicago, USA |
| p-PI3K Tyr458 | 1:1000 | / | Rabbit Polyclonal | Proteintech Group Chicago, USA |
| Akt | 1:1000 | / | Rabbit Polyclonal | Proteintech Group Chicago, USA |
| p-Akt Ser473 | 1:1000 | / | Rabbit Polyclonal | Proteintech Group Chicago, USA |
| mTOR | 1:1000 | / | Rabbit Polyclonal | Proteintech Group Chicago, USA |
| p-mTOR Ser2448 | 1:1000 | / | Rabbit Polyclonal | Proteintech Group Chicago, USA |

**Supplementary Table 4. siRNA sequence used in this study**

| **siRNA** | **sense sequence** |
| --- | --- |
| siRNA-DPEP1-1 | 5’TGGCAGCTGCTGGATATGTTCAACA3’ |
| siRNA-DPEP1-2 | 5’CCGACCATCTGGATCACATCAAGGA3’ |
| siRNA-DPEP1-3 | 5’GGGACTTTGATGGTGTTCCAAGGGT3’ |
| siRNA-NC | 5’CCAGUUUACCUAACGCAAUTT3’ |
