# Supplementary material for: DPEP1 is a direct target of miR-193a-5p and promotes hepatoblastoma progression by PI3K/Akt/mTOR pathway
Source: Cell Death Dis. 2019 Sep 20;10(10):701. doi: 10.1038/s41419-019-1943-0 (PMC6754441; doi:10.1038/s41419-019-1943-0)
Supplement: Supplementary file 2 — Supplementary Figure legends. [file 41419_2019_1943_MOESM2_ESM.docx]

**Fig S1. Knockdown of DPEP1 suppresses HB cell proliferation, migration and invasion. a** Cell proliferation of HepG2 or Huh-6 cells transfected with NC or DPEP1 siRNA2 was analyzed by EDU staining assay. Scale bars, 50μm. **b** Colony formation of HepG2 or HuH-6 cells transfected with NC or DPEP1 siRNA2. Scale bars, 8mm. **c** The migration capability of HepG2 or HuH-6 cells transfected with NC or DPEP1 siRNA2 was analyzed by wound-healing assay at indicated time points. Scale bars, 500μm. **d** The invasion capability of HepG2 or HuH-6 cells transfected with NC or DPEP1 siRNA2 was analyzed by transwell assay. **e** Cell proliferation of HepG2 or Huh-6 cells transfected with NC or DPEP1 siRNA3 was analyzed by EDU staining assay. Scale bars, 50μm. **f** Colony formation of HepG2 or HuH-6 cells transfected with NC or DPEP1 siRNA3. Scale bars, 8mm. **g** The migration capability of HepG2 or HuH-6 cells transfected with NC or DPEP1 siRNA3 was analyzed by wound-healing assay at indicated time points. Scale bars, 500μm. **h** The invasion capability of HepG2 or HuH-6 cells transfected with NC or DPEP1 siRNA3 was analyzed by transwell assay. Scale bars, 50μm. **p* < 0.05.

**Fig S2. Overexpression of DPEP1 promotes HB cell proliferation, colony formation, migration and invasion *in vitro*.** HepG2 or HuH-6 cells were transfected with negative control (NC), DPEP1 overexpressing plasmid or left untreated (Blank). **a** The protein levels of DPEP1 in different groups were analyzed by western blot. **b, c** Cell proliferation of HepG2 or HuH-6 cells was analyzed by CCK-8 assay. **d** Cell proliferation of HepG2 or HuH-6 cells was analyzed by EDU staining assay. Scale bars, 50μm. **e** Colony formation of HepG2 or HuH-6 cells. Scale bars, 8mm. **f** The migration capability of HepG2 or HuH-6 cells was analyzed by wound-healing assay at indicated time points. Scale bars, 500μm. **g** The invasion capability of HepG2 or HuH-6 cells was analyzed by transwell assay. Scale bars, 50μm. **p* < 0.05, ***p* < 0.01.

**Fig. S3 Exploration of DPEP1 action pathway.** **a** Heatmap of differential gene expression analysis generated using GEO HB data. **b** Expression levels of PI3K, p-PI3K(Tyr458), Akt, p-Akt (Ser473), mTOR, and p-mTOR (Ser2448) in HuH-6 transfected with DPEP1 siRNA, NC siRNA, LY294002, or DMSO were analyzed by western blot. **c** Expression levels of PI3K, p-PI3K(Tyr458), Akt, p-Akt (Ser473), mTOR, and p-mTOR (Ser2448) in HuH-6 transfected with DPEP1 plasmid, NC plasmid, LY294002, or DMSO were analyzed by western blot. **d** Cell colony formation (up panel) and invasion (lower panel) of HepG2 cells transfected with DPEP1 siRNA or Negative control, DPEP1 plasmid or negative control, or cultured with DMSO or LY294002. The representative result of at least three independent experiments was shown. Cell colony formation: Scale bars, 8mm. Invasion: Scale bars, 50μm.

**Fig. S4 DPEP1** **downregulation inhibits PI3K/Akt/mTOR signaling. a** Expression levels of PI3K, p-PI3K(Tyr458), Akt, p-Akt (Ser473), mTOR, and p-mTOR (Ser2448) in HepG2 transfected with DPEP1 siRNA, NC siRNA, DMSO or IGF-1 were analyzed by western blot. **b** Cell colony formation (up panel) and invasion (lower panel) of HepG2 cells treated with DPEP1 siRNA, Negative control, DMSO or IGF-1.

**Fig. S5 The correlation between DPEP1 and** **Wnt/β-catenin signaling. a-c** The Gene Set Enrichment Analysis (GSEA) disclosed the correlation between DPEP1 and Wnt/β-catenin signaling. **b** Expression levels of β-catenin, APC, cyclinD1 and c-myc in HepG2 and HuH-6 transfected with DPEP1, DPEP1 siRNA, or cells only (Blank) were analyzed by western blot. **d** Expression levels of GLUL in HepG2 and HuH-6 transfected with DPEP1, DPEP1 siRNA, or NC were analyzed by western blot.
