# Supplementary figures and images for: DPEP1 is a direct target of miR-193a-5p and promotes hepatoblastoma progression by PI3K/Akt/mTOR pathway
Source: Cell Death Dis. 2019 Sep 20;10(10):701. doi: 10.1038/s41419-019-1943-0 (PMC6754441; doi:10.1038/s41419-019-1943-0)

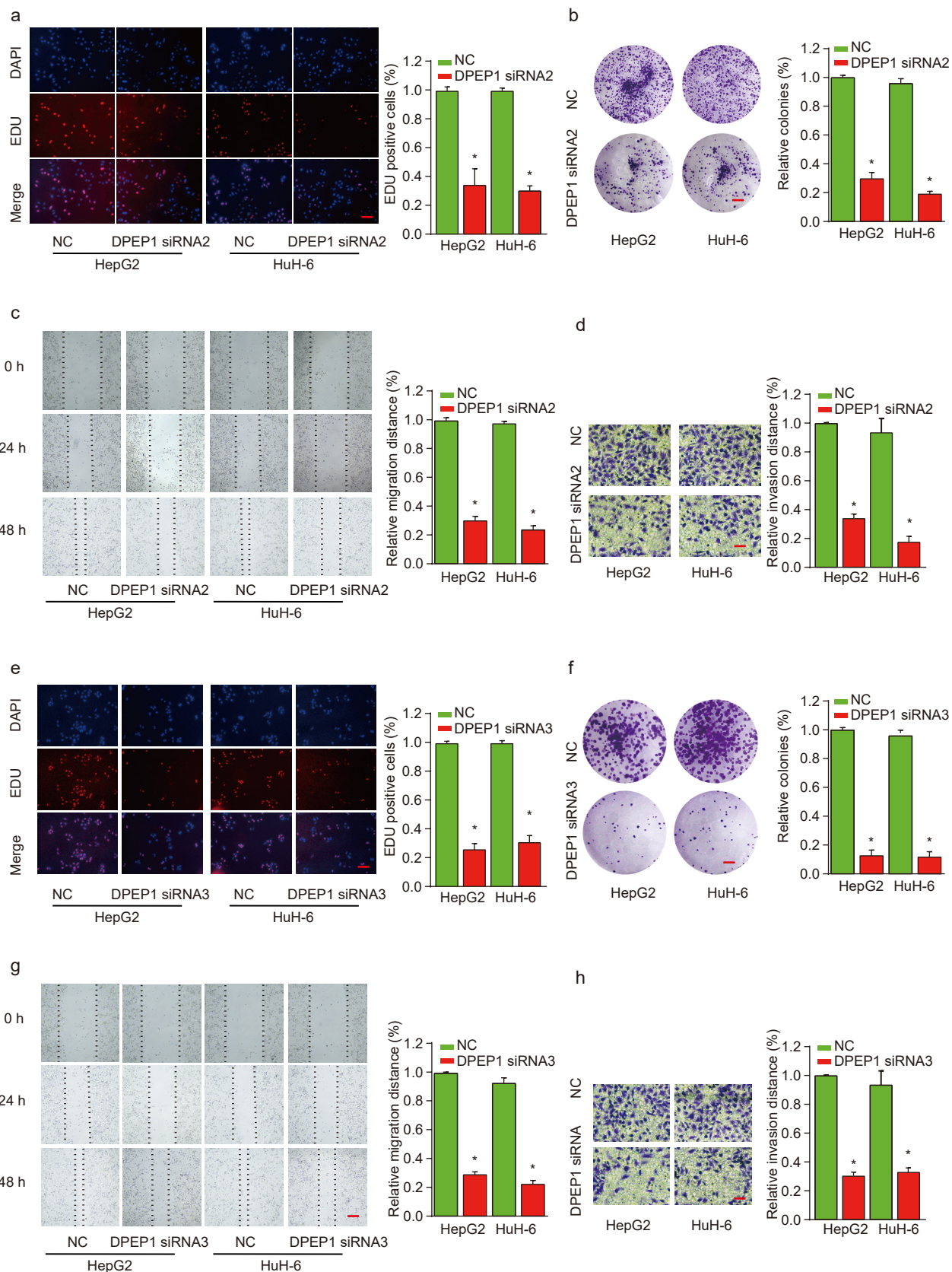

Supplement: Supplementary file 3 — Supplementary Figure S1. [file 41419_2019_1943_MOESM3_ESM.pdf]

a

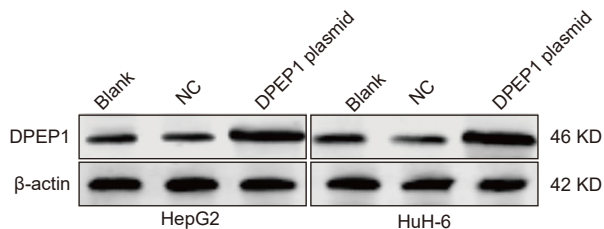

b

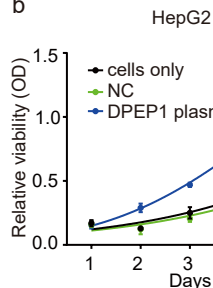

c

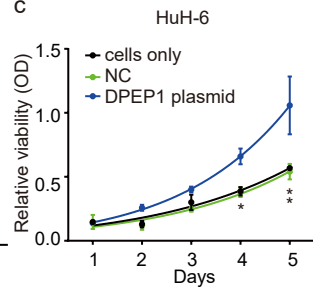

d

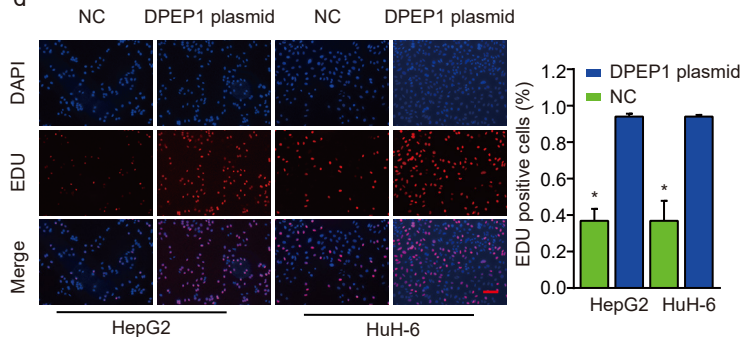

e

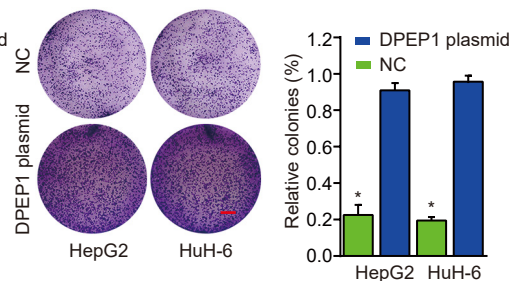

f

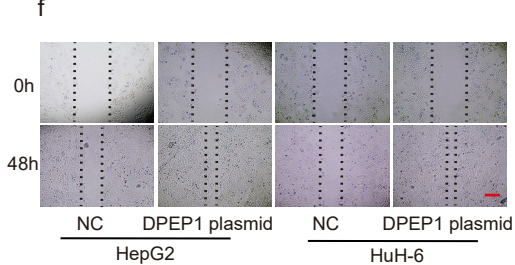

g

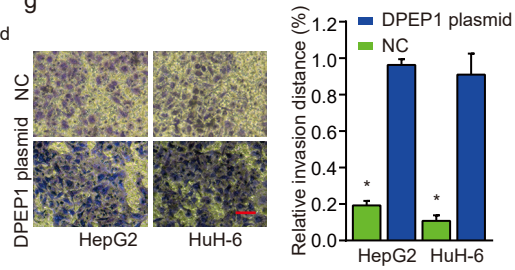

Supplement: Supplementary file 4 — Supplementary Figure S2. [file 41419_2019_1943_MOESM4_ESM.pdf]

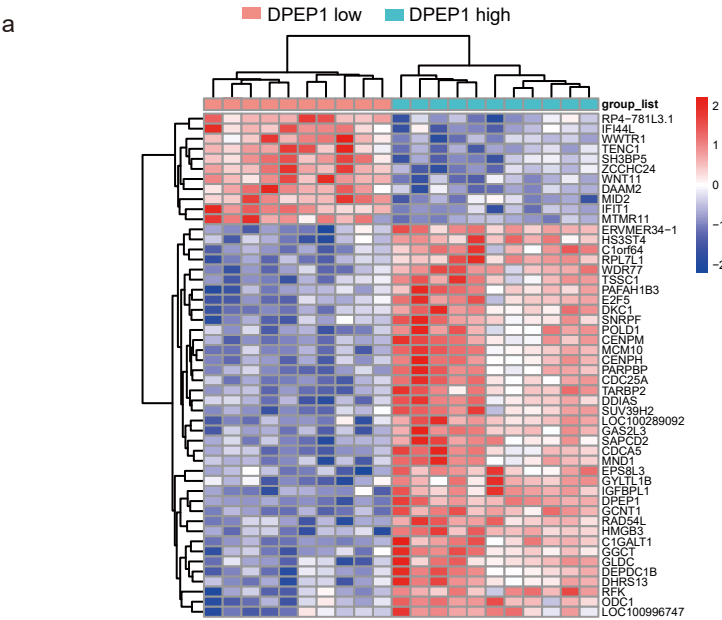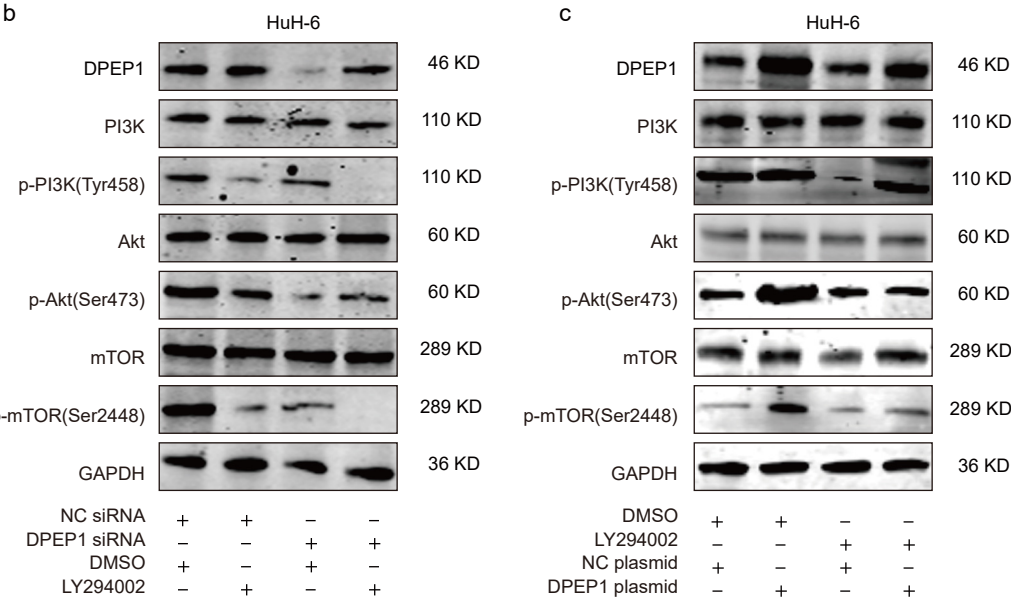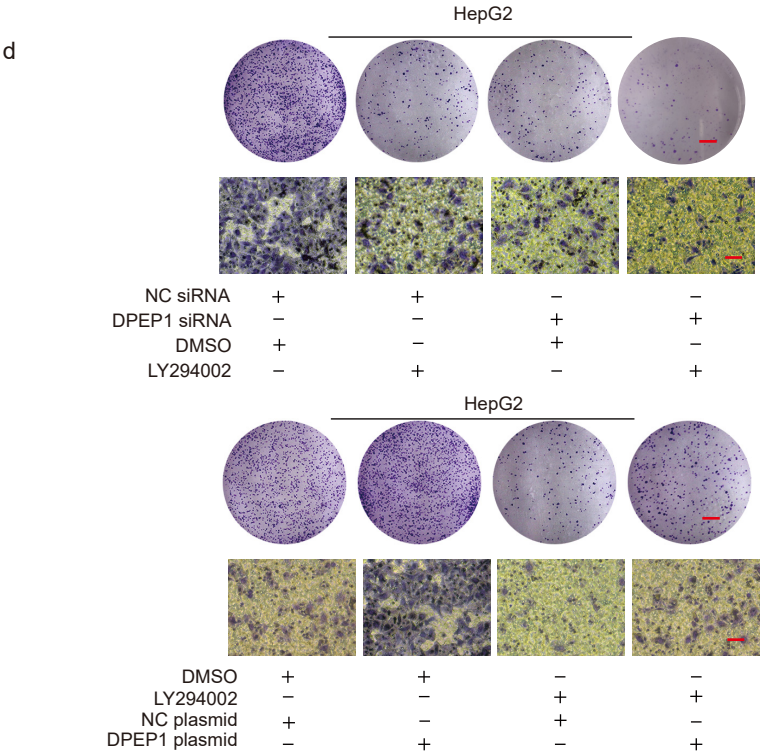

Supplement: Supplementary file 5 — Supplementary Figure S3. [file 41419_2019_1943_MOESM5_ESM.pdf]

a

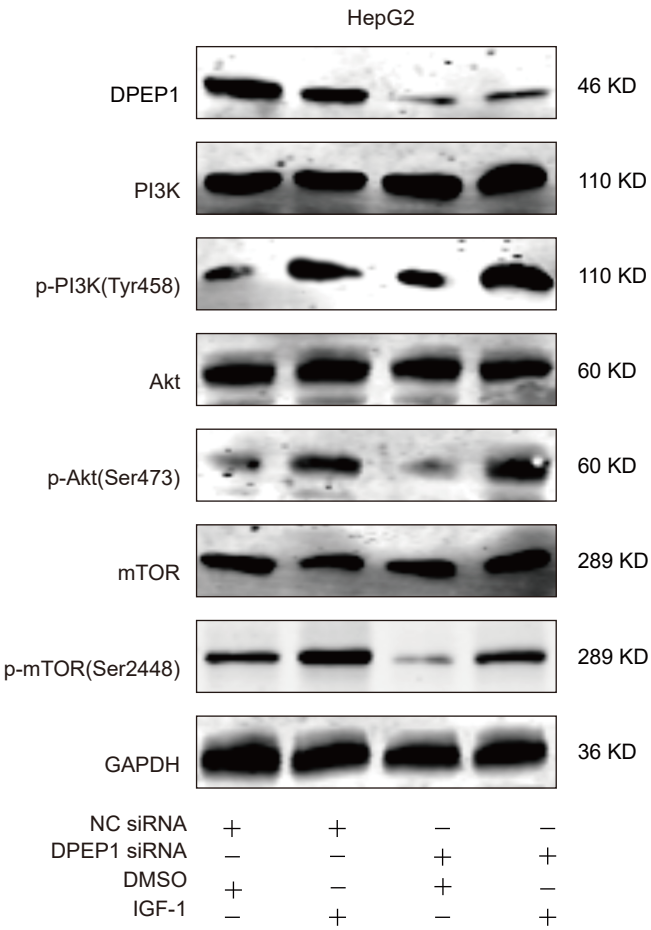

b

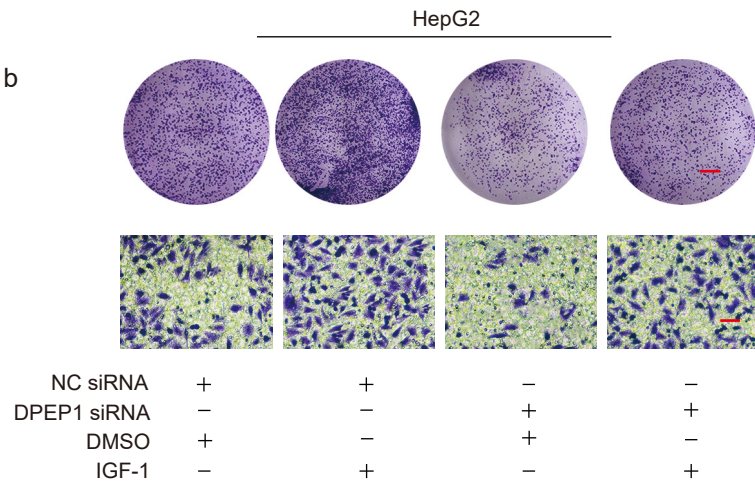

Supplement: Supplementary file 6 — Supplementary Figure S4. [file 41419_2019_1943_MOESM6_ESM.pdf]

a

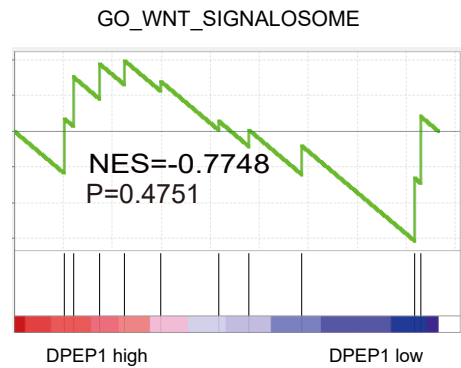

b

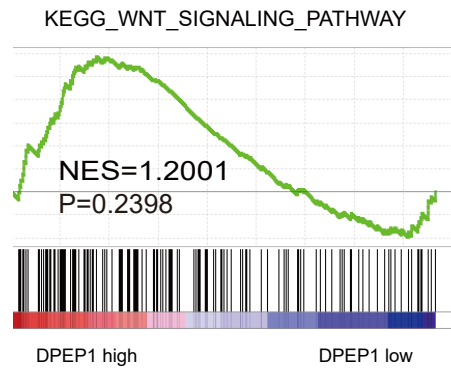

c

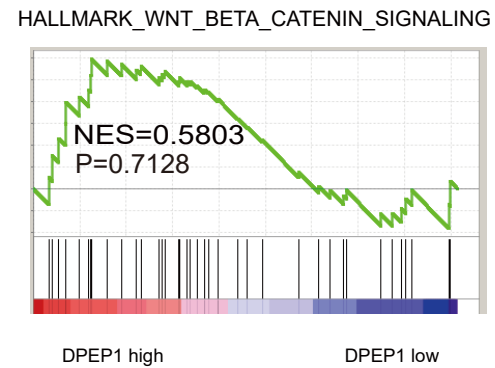

d

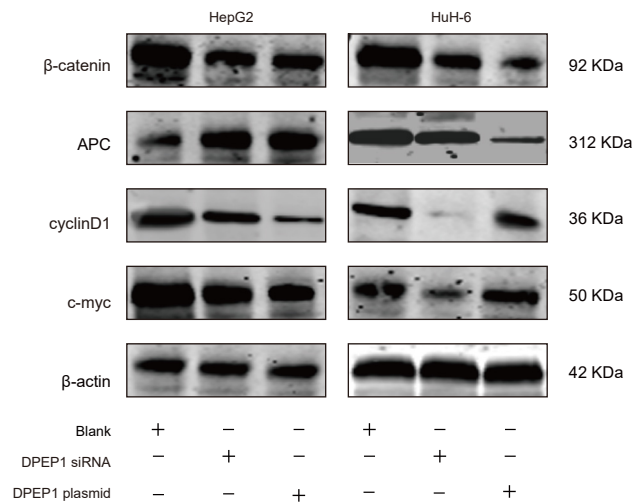

e

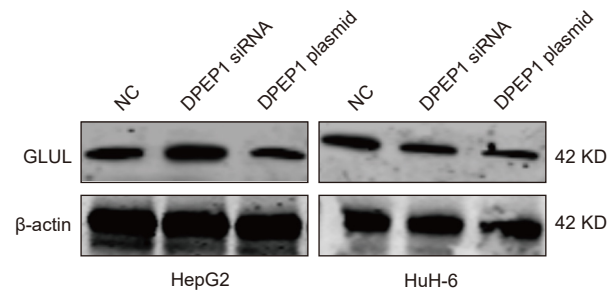

Supplement: Supplementary file 7 — Supplementary Figure S5. [file 41419_2019_1943_MOESM7_ESM.pdf]
