# Supplementary material for: DPEP1 is a direct target of miR-193a-5p and promotes hepatoblastoma progression by PI3K/Akt/mTOR pathway
Source: Cell Death Dis. 2019 Sep 20;10(10):701. doi: 10.1038/s41419-019-1943-0 (PMC6754441; doi:10.1038/s41419-019-1943-0)

Cell Line Authentication – STR Profiling

Sample Type:Cell Line

Sample from:The First Affiliated Hospital of Zhengzhou University, Zhengzhou 450052, China

Testing Method: STR Genotyping

Report Time: August 10, 2019

Sample code

Table 1. Sample Code

Customer's code	Company Code
01	20190810-03-01

Sample Number:1

Sample Type: Cell line

Testing Type: STR

Sample From:The First Affiliated Hospital of Zhengzhou University

Testing Method:

DNA was extracted by a commercial kit from CORNING (AP-EMN-BL-GDNA-250G). The twenty STRs including Amelogenin locus were amplified by six multiplex PCR and separated on ABI 3730XL Genetic Analyzer. The signals were then analyzed by the software GeneMapper.

Data Interpretation:

Cell lines were authenticated using Short Tandem Repeat (STR) analysis as described in 2012 in

ANSI Standard (ASN-0002) by the ATCC Standards Development Organization (SDO) and in

Capes-Davis et al., Match criteria for human cell line authentication: Where do we draw the line?

Int J Cancer.2013;132(11):2510-9.

Test Results:

1. Result

Table 2. Matching information on the cell lines

Sample Code	Multi-allele	Cell line matched	Cell Bank	Percentage
20190810-03-01	NO	HEPG2	ATCC	9/9

- **Multi-allele means some STR contain more than two loci.**

2. Sample Description

20190810-03 The DNA of the cell lines found to basic match the type of cell lines in a cell lineretrieval, ATCC database shows that cells called HEPG2 corresponding to the cell number CRL-11997 . No multiple alleles were found in this cell line.

3. Genotyping Result

STR and Amelogenin Genotyping Results of Cell line 20171012-01						
Loci	**Sample**：8			**Cell Bank information**：HEPG2		
	Allele1	Allele2	Allele3	Allele1	Allele2	Allele3
D5S818	11	12	13	11	12	
D13S317	9	13		9	13	
D7S820	10	10		10	10	
D16S539	12	13		12	13	
VWA	17	17		17	17	

TH01	9	9		9	9	
AMEL	X	Y		X	Y	
TPOX	8	9		8	9	
CSF1PO	10	11		10	11	
D12S391	21	25				
FGA	22	25				
D2S1338	19	20				
D21S11	29	30	31			
D18S51	13	14				
D8S1179	15	16				
D3S1358	15	16				
D6S1043	13	13				
PENTAE	15	20				
D19S433	15.2	15.2				
PENTAD	9	13				

Others

1. Genotyping Strategy and Site Distribution

Attached Table. Experimental Strategy and Sites

	**Strategy 1**	**Strategy 2**	**Strategy 3**	**Strategy 4**
1	TH01	TPOX	D3S1358	AMEL

2	D12S391	VWA	D13S317	D5S818
3	D7S820	D8S1179	D6S1043	D2S1338
4	CSF1PO	PENTAD	D16S539	D21S11
5	FGA		D19S433	D18S51
6	PENTAE			

The allele match algorithm compares the 8 core loci plus amelogenin only, even though alleles from all loci will be reported when available.

2. DSMZ tools was used to carry on the cell line comparison, which contains 2455 cell lines STR data from ATCC, DSMZ, JCRB ,ECACC and RIKEN databases. If the cell is not included in the above cell library, users need to compared with other databases.

Cell Line Authentication 1012 16 18

Sample File	Sample Name	Panel	OS	SQ
02_D02.fsa	8	STR Profile 1-human		

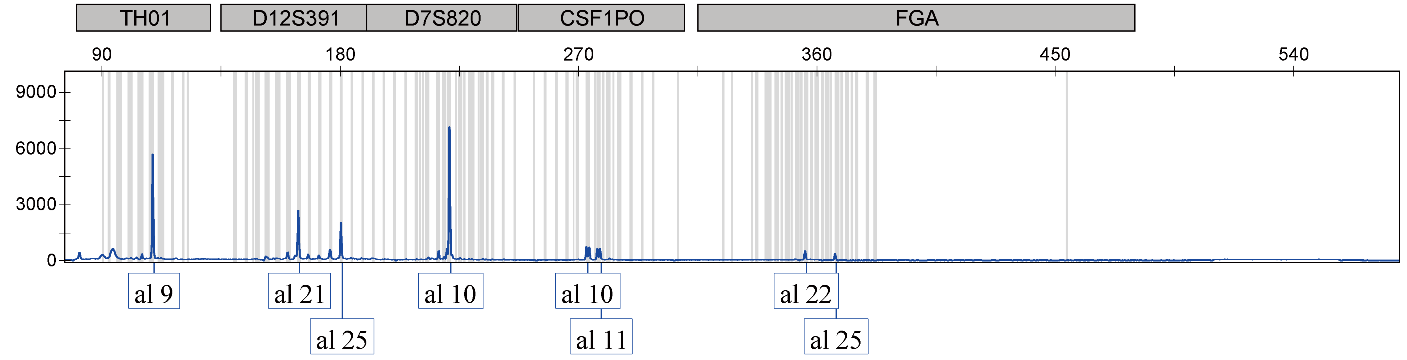

Sample File	Sample Name	Panel	OS	SQ
02_D02.fsa	8	STR Profile 1-human		

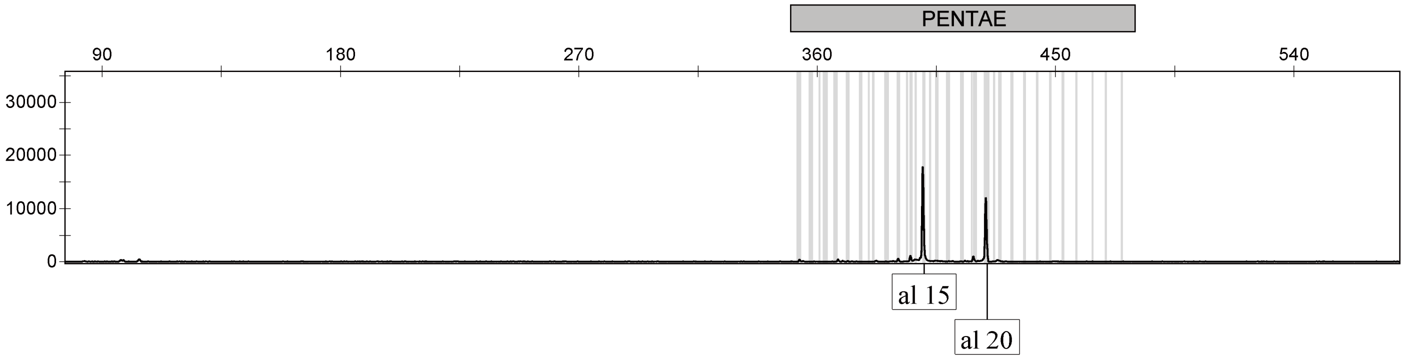

Sample File	Sample Name	Panel	OS	SQ
11_D11.fsa	8	STR Profile 2-human		

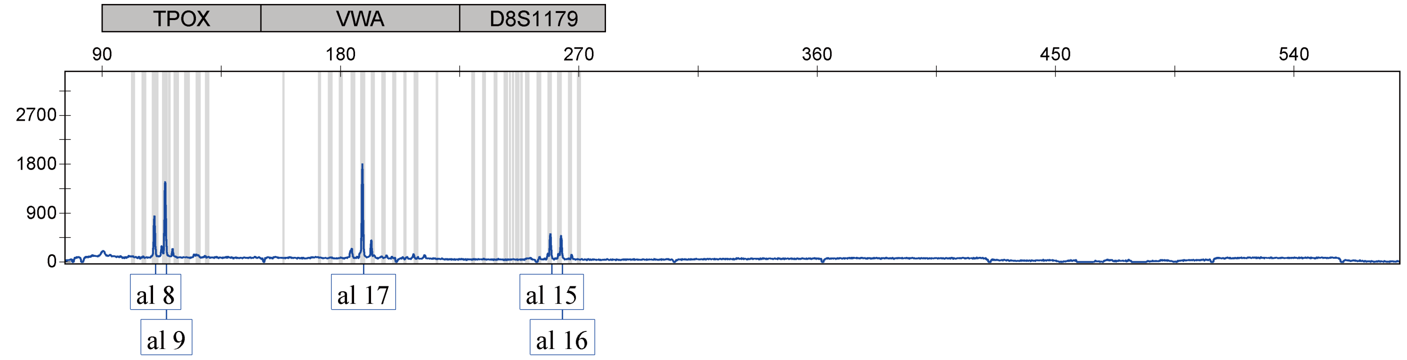

Applied Biosystems

GeneMapper 4.0

Cell Line Authentication 1012 16 18

Sample File	Sample Name	Panel	OS	SQ
11_D11.fsa	8	STR Profile 2-human		

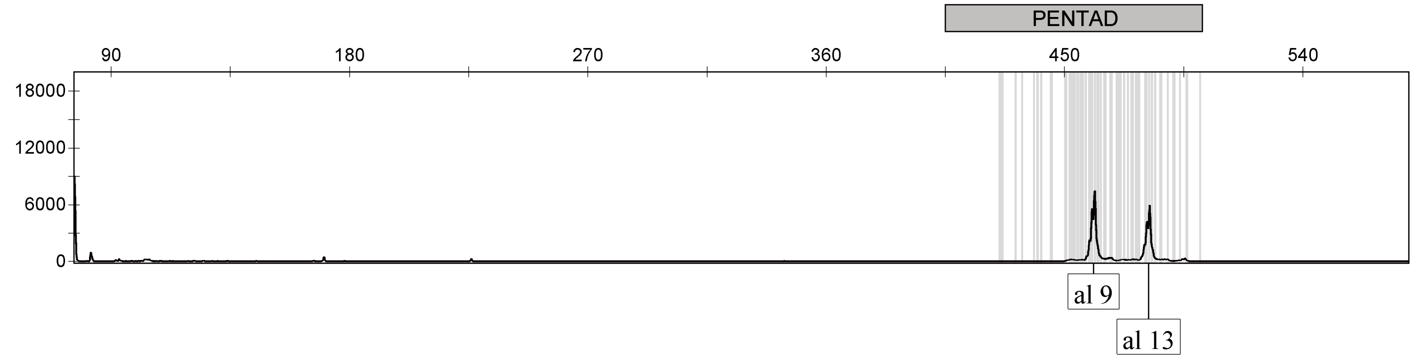

Sample File	Sample Name	Panel	OS	SQ
08_D08.fsa	8	STR Profile 3-human		

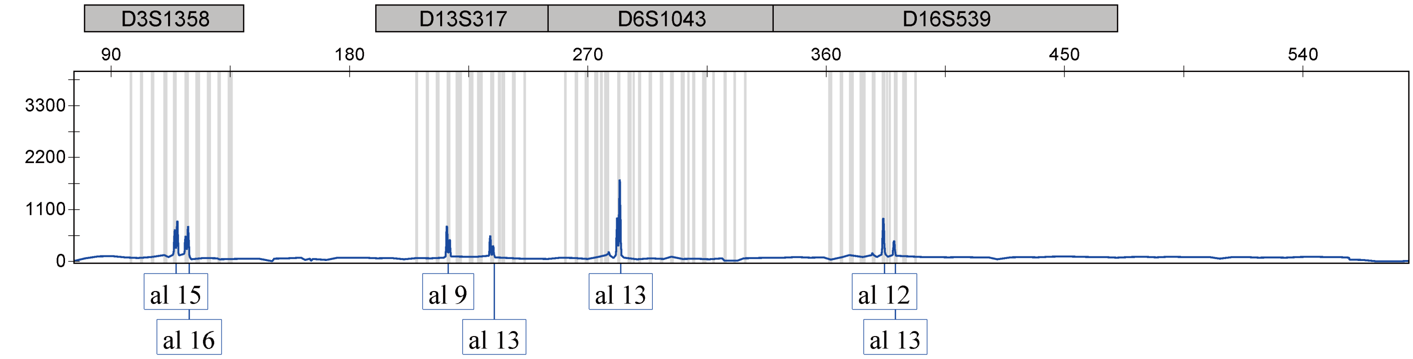

Sample File	Sample Name	Panel	OS	SQ
08_D08.fsa	8	STR Profile 3-human		

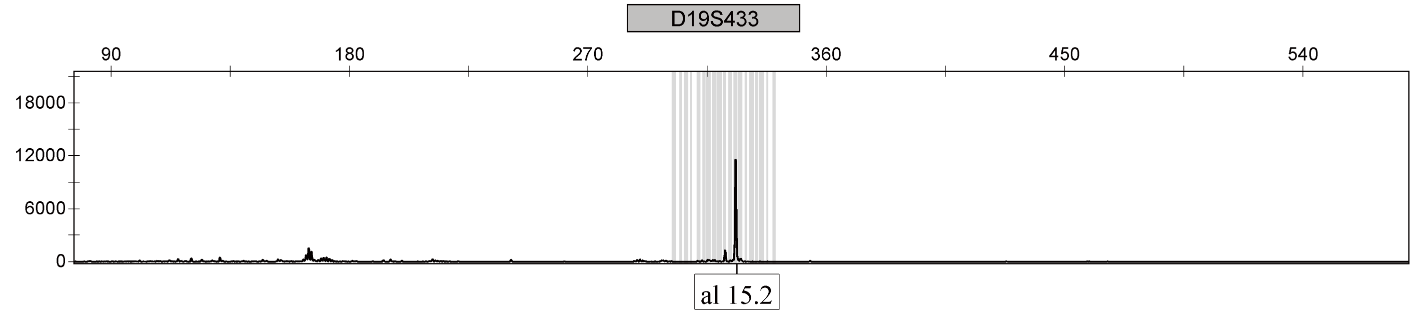

Applied Biosystems

GeneMapper 4.0

Cell Line Authentication 1012 16 18

Sample File	Sample Name	Panel	OS	SQ
05_D05.fsa	8	STR Profile 4-human		

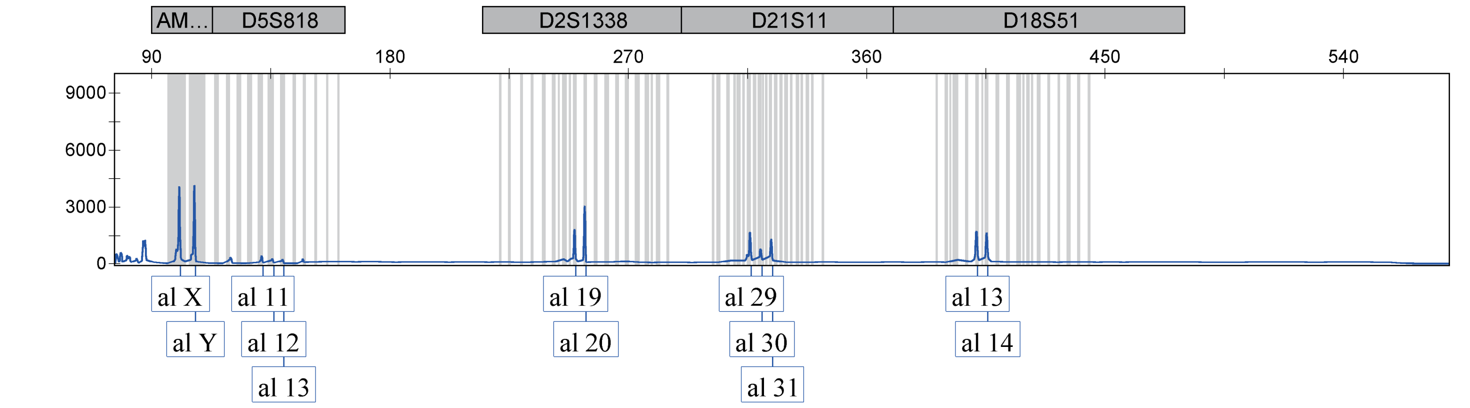

05_D05.fsa	8	STR Profile 4-human		

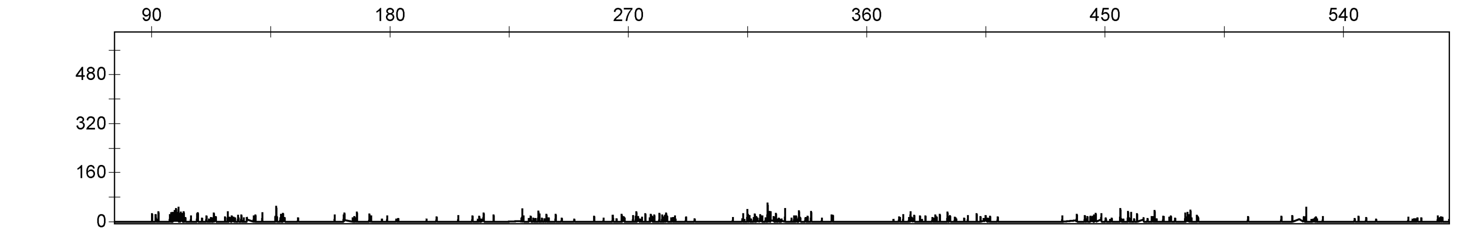

Supplement: Supplementary file 8 — hepg2-STR Profiling [file 41419_2019_1943_MOESM8_ESM.pdf]
