# Supplementary material for: DPEP1 is a direct target of miR-193a-5p and promotes hepatoblastoma progression by PI3K/Akt/mTOR pathway
Source: Cell Death Dis. 2019 Sep 20;10(10):701. doi: 10.1038/s41419-019-1943-0 (PMC6754441; doi:10.1038/s41419-019-1943-0)

Cell Line Authentication – STR Profiling

Sample Type:Cell Line

Sample from:The First Affiliated Hospital of Zhengzhou University, Zhengzhou 450052, China

Testing Method: STR Genotyping

Report Time: August 10, 2019

Cell Line Authentication – STR Profiling Report

Sample code

Table 1. Sample Code

Customer's code	Company Code
2	20190810-03

Sample Number:1

Sample Type: Cell line

Testing Type: STR
Sample From: The First Affiliated Hospital of Zhengzhou University

Testing Method:

DNA was extracted by a commercial kit from CORNING (AP-EMN-BL-GDNA-250G). The twenty STRs including Amelogenin locus were amplified by six multiplex PCR and separated on ABI 3730XL Genetic Analyzer. The signals were then analyzed by the software GeneMapper.

Data Interpretation:

Cell lines were authenticated using Short Tandem Repeat (STR) analysis as described in 2012 in ANSI Standard (ASN-0002) by the ATCC Standards Development Organization (SDO) and in Capes-Davis et al., Match criteria for human cell line authentication: Where do we draw the line? Int J Cancer.2013;132(11):2510-9.

Test Results:

1. Result

Table 2. Matching information on the cell lines

Sample Code	Multi-allele	Cell line matched	Cell Bank	Percentage
20190810-03-02	NO	HELA(CHANG LIVER)	DSMZ	9/9

- **Multi-allele means some STR contain more than two loci.**

2. Sample Description

20190810-03-02 The DNA of the cell lines found to basic match the type of cell lines in a cell line

STR and Amelogenin Genotyping Results of Cell line 20190810-03-02						
Loci	Sample:2			Cell Bank information： HELA (Changliver)		
	Allele1	Allele2	Allele3	Allele1	Allele2	Allele3
D5S818	11	12		11	12	
D13S317	12	13.3		12	13.3	
D7S820	8	12		8	12	
D16S539	9	10		9	10	
VWA	16	18		16	18	
TH01	7	7		7	7	
AMEL	X	X		X	X	
TPOX	8	12		8	12	
CSF1PO	9	10		9	10	
D12S391	20	25				
FGA	21	21				
D2S1338	17	17				
D21S11	27	28				
D18S51	16	16				
D8S1179	12	13				
D3S1358	15	18				
D6S1043	18	19				
PENTAE	7	17				
D19S433	13	14				
PENTAD	8	15				

Others:

1. Genotyping Strategy and Site Distribution

Attached Table. Experimental Strategy and Sites

	**Strategy 1**	**Strategy 2**	**Strategy 3**	**Strategy 4**
1	TH01	TPOX	D3S1358	AMEL
2	D12S391	VWA	D13S317	D5S818
3	D7S820	D8S1179	D6S1043	D2S1338
4	CSF1PO	PENTAD	D16S539	D21S11
5	FGA		D19S433	D18S51
6	PENTAE			

Applied Biosystems

GeneMapper 4.0

Cell Line Authentication 0920

Sample File	Sample Name	Panel	OS	SQ
01_A01.fsa	2	STR Profile 1-human		

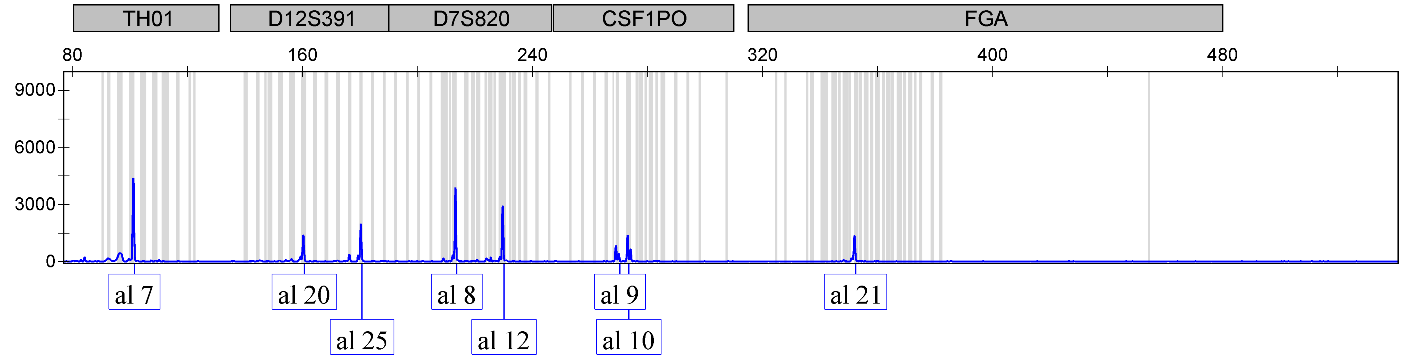

Sample File	Sample Name	Panel	OS	SQ
01_A01.fsa	2	STR Profile 1-human		

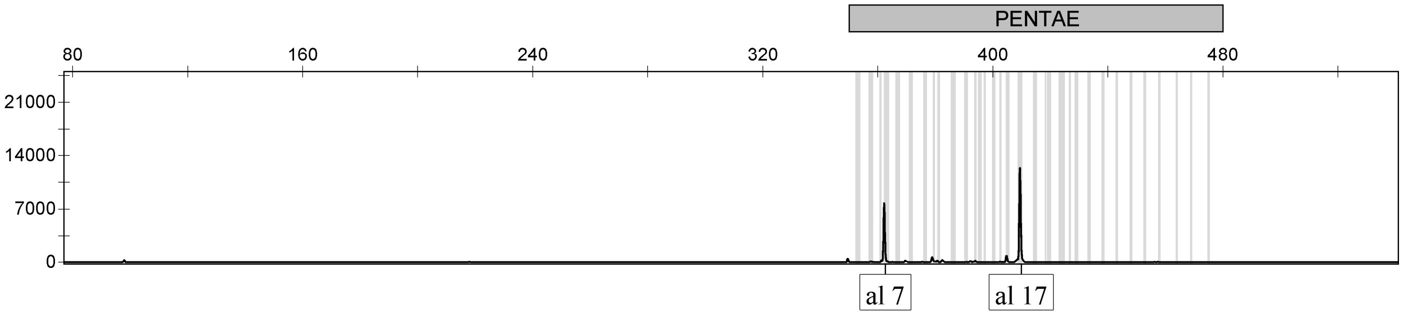

Sample File	Sample Name	Panel	OS	SQ
25_A04.fsa	2	STR Profile 2-human		

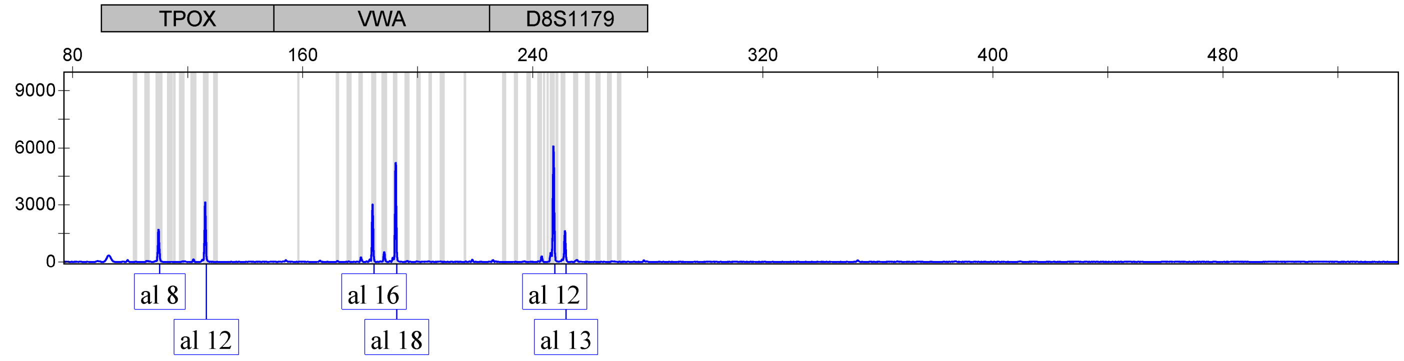

GeneMapper 4.0

Cell Line Authentication 0920

Sample File	Sample Name	Panel	OS	SQ
25 A04.fsa	2	STR Profile 2-human		

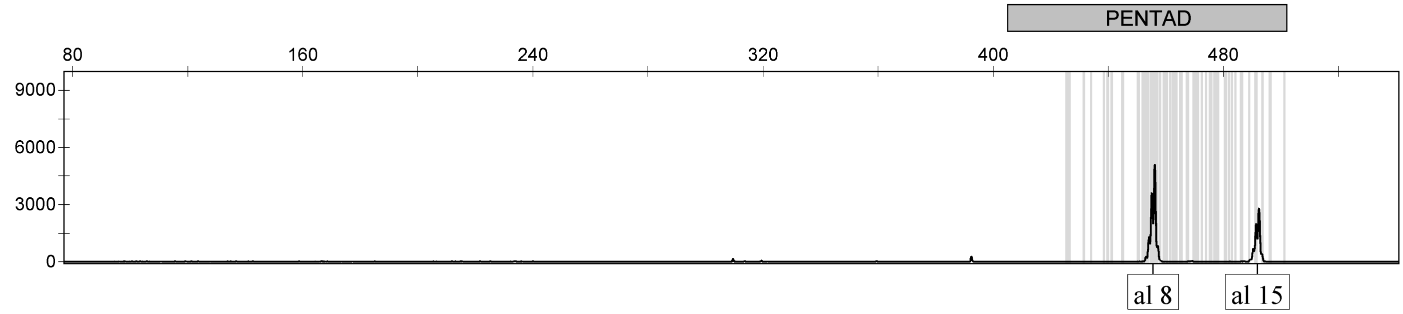

41 A06.fsa	2	STR Profile 3-human		

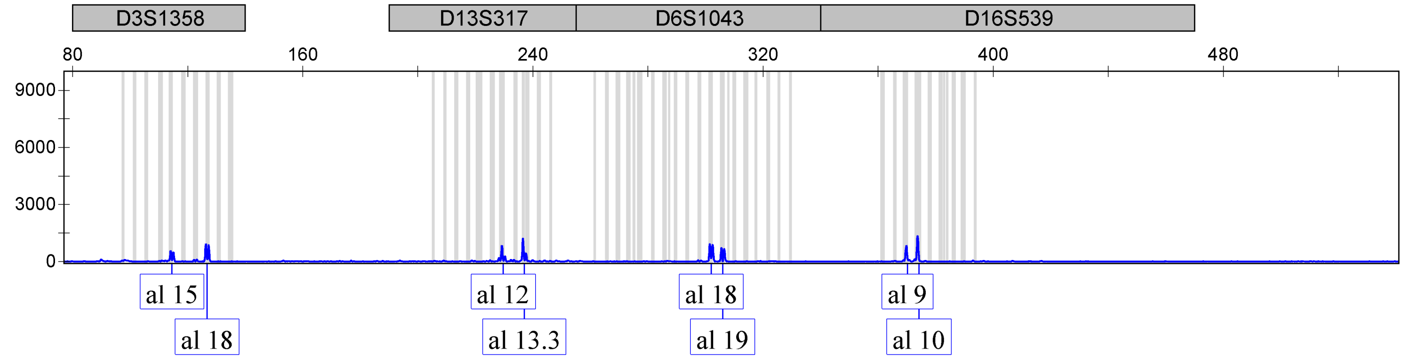

41 A06.fsa	2	STR Profile 3-human		

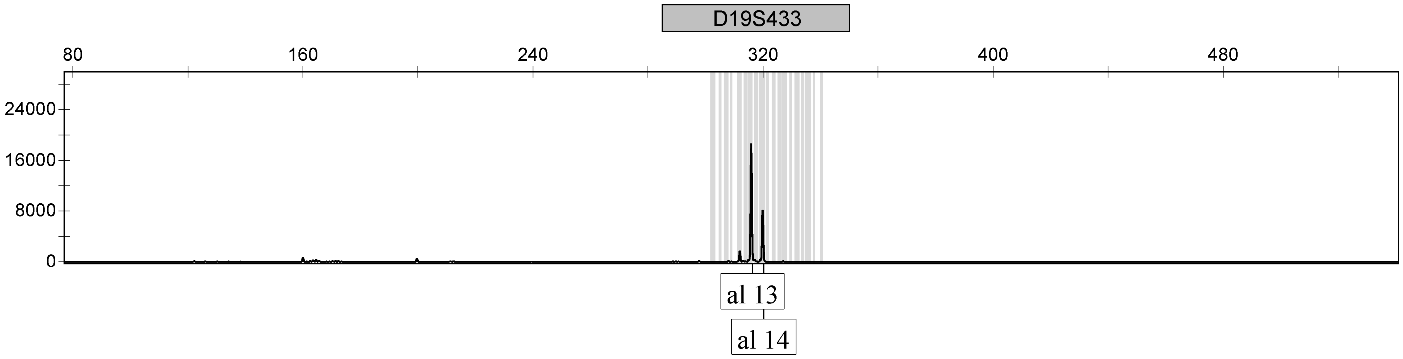

Applied Biosystems

GeneMapper 4.0

Sample File	Sample Name	Panel	OS	SQ
57_A08.fsa	2	STR Profile 4-human		

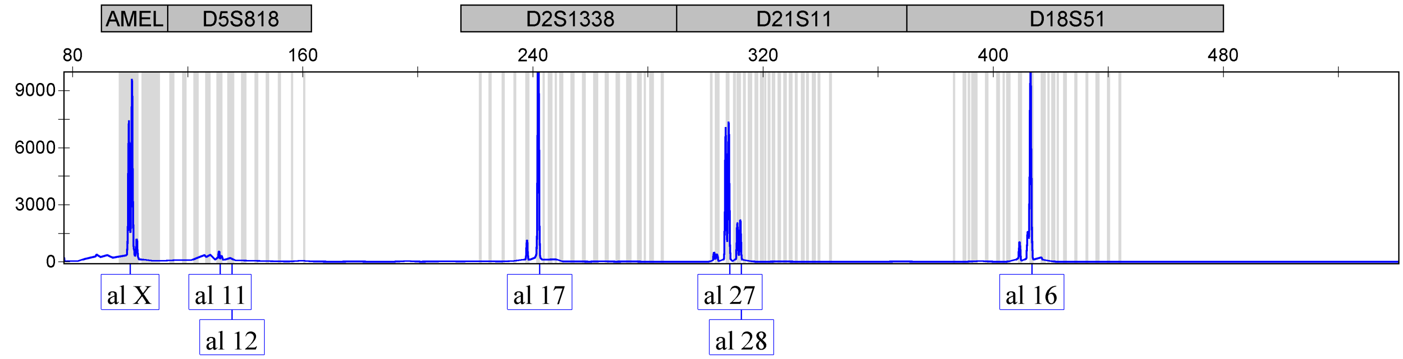

57_A08.fsa	2	STR Profile 4-human		

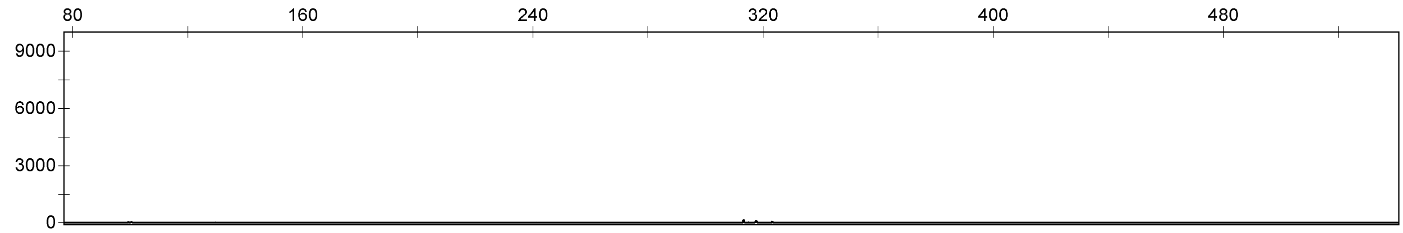

Supplement: Supplementary file 9 — CHANG LIVER-STR Profiling [file 41419_2019_1943_MOESM9_ESM.pdf]
