# Supplementary material for: DPEP1 is a direct target of miR-193a-5p and promotes hepatoblastoma progression by PI3K/Akt/mTOR pathway
Source: Cell Death Dis. 2019 Sep 20;10(10):701. doi: 10.1038/s41419-019-1943-0 (PMC6754441; doi:10.1038/s41419-019-1943-0)

Cell Line Authentication – STR Profiling

Sample Type:Cell Line

Sample from:The First Affiliated Hospital of Zhengzhou University, Zhengzhou 450052, China

Testing Method: STR Genotyping

Report Time: August 10, 2019

Cell Line Authentication – STR Profiling Report

Sample code

Table 1. Sample Code

Customer's code	Company Code
3	20190810-03

Sample Number: 1

Sample Type: Cell line

Testing Type: STR

Sample From: The First Affiliated Hospital of Zhengzhou University

Testing Method:

DNA was extracted by a commercial kit from CORNING (AP-EMN-BL-GDNA-250G). The twenty STRs including Amelogenin locus were amplified by six multiplex PCR and separated on ABI 3730XL Genetic Analyzer. The signals were then analyzed by the software GeneMapper.

Data Interpretation:

Cell lines were authenticated using Short Tandem Repeat (STR) analysis as described in 2012 in ANSI Standard (ASN-0002) by the ATCC Standards Development Organization (SDO) and in Capes-Davis et al., Match criteria for human cell line authentication: Where do we draw the line? Int J Cancer.2013;132(11):2510-9.

Test Results:

1. Result

Table 2. Matching information on the cell lines

Sample Code	Multi-allele	Cell line matched	Cell Bank	Percentage
20190810-03-03	NO	HUH-6	DSMZ	9/9

- **Multi-allele means some STR contain more than two loci.**

2. Sample Description

20190810-03-03 The DNA of the cell lines found to basic match the type of cell lines in a cell line

retrieval, DSMZ database shows that cells called **HUH-6** corresponding to the cell number **JCRB0401**. No multiple alleles were found in this cell line.

3. Genotyping Result

STR and Amelogenin Genotyping Results of Cell line 20190810-03-03						
Loci	**Sample**：3			**Cell Bank information**：HuH-6		
	Allele1	Allele2	Allele3	Allele1	Allele2	Allele3
D5S818	12	12		12	12	
D13S317	10	11		10	11	
D7S820	11	11		11	11	
D16S539	10	10		10	10	
VWA	16	18		16	18	
TH01	7	7		7	7	
AMEL	X	Y		X	X	
TPOX	8	11		8	11	
CSF1PO	11	11		11	11	
D12S391	20	21				
FGA	22	24				
D2S1338	19	19				
D21S11	30	30				
D18S51	15	15				
D8S1179	14	15				
D3S1358	15	15				
D6S1043	13	15				
PENTAE	11	11				

D19S433	13	14				
PENTAD	12	12				

Others:

1. Genotyping Strategy and Site Distribution

Attached Table. Experimental Strategy and Sites

	**Strategy 1**	**Strategy 2**	**Strategy 3**	**Strategy 4**	**Strategy 5**	**Strategy 6**
1	TH01	AMEL	TPOX	D3S1358	PENTAE	D19S433
2	D12S391	D5S818	VWA	D13S317		PENTAD
3	D7S820	D2S1338	D8S1179	D6S1043		
4	CSF1PO	D21S11		D16S539		
5	FGA	D18S5				

The allele match algorithm compares the 8 core loci plus amelogenin only, even though alleles from all loci will be reported when available.

2. DSMZ tools was used to carry on the cell line comparison, which contains 2455 cell lines STR data from ATCC, DSMZ, JCRB ,ECACC and RIKEN databases. If the cell is not included in the above cell library, users need to compared with other databases.

Applied Biosystems

GeneMapper 4.0

Cell Line Authentication 0920

Sample File	Sample Name	Panel	OS	SQ
02_B01.fsa	3	STR Profile 1-human		

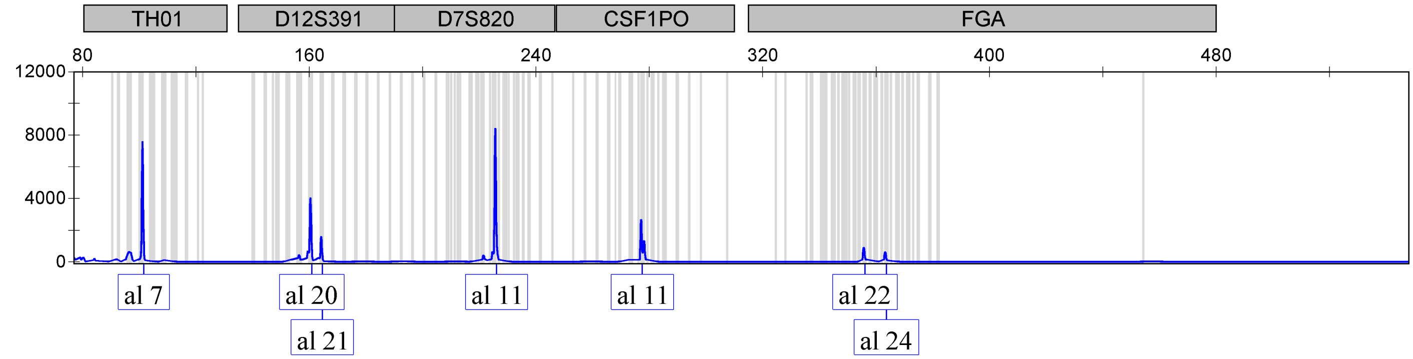

Sample File	Sample Name	Panel	OS	SQ
02_B01.fsa	3	STR Profile 1-human		

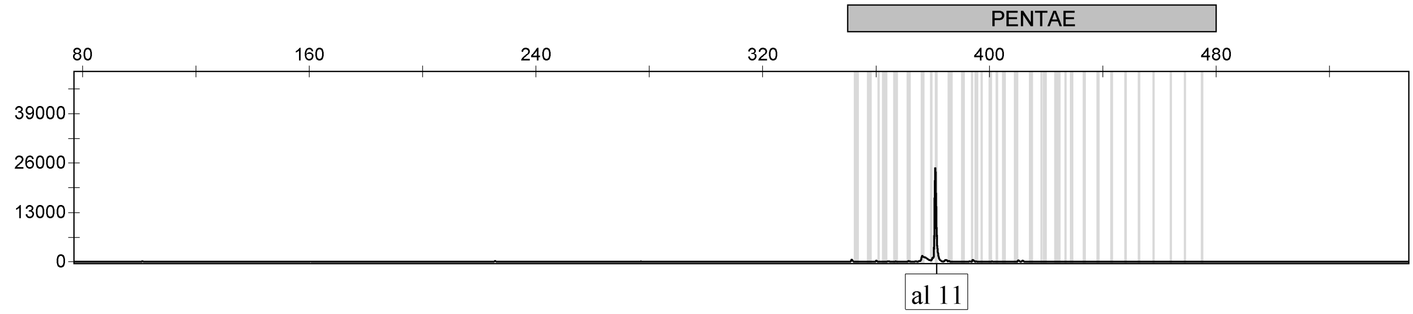

Sample File	Sample Name	Panel	OS	SQ
26_B04.fsa	3	STR Profile 2-human		

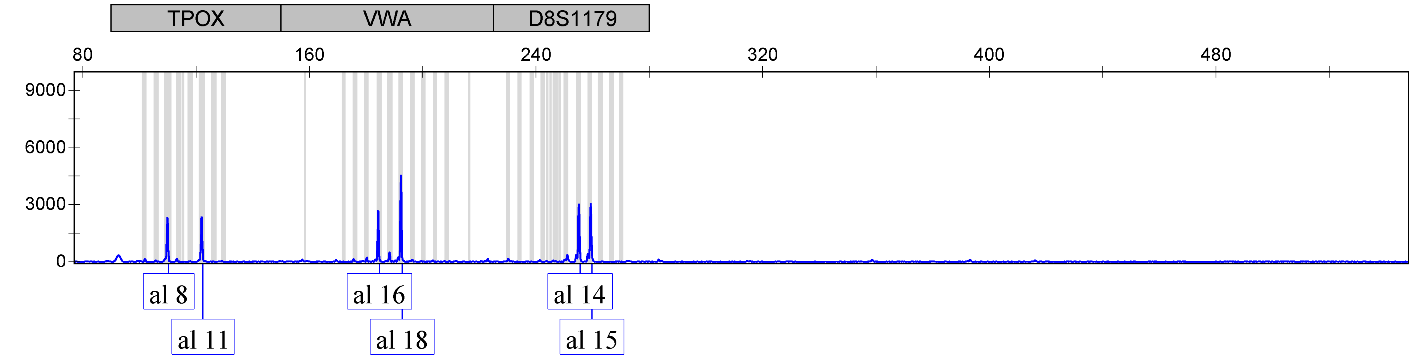

Applied Biosystems
GeneMapper 4.0

Cell Line Authentication 0920

Sample File	Sample Name	Panel	OS	SQ
26_B04.fsa	3	STR Profile 2-human		

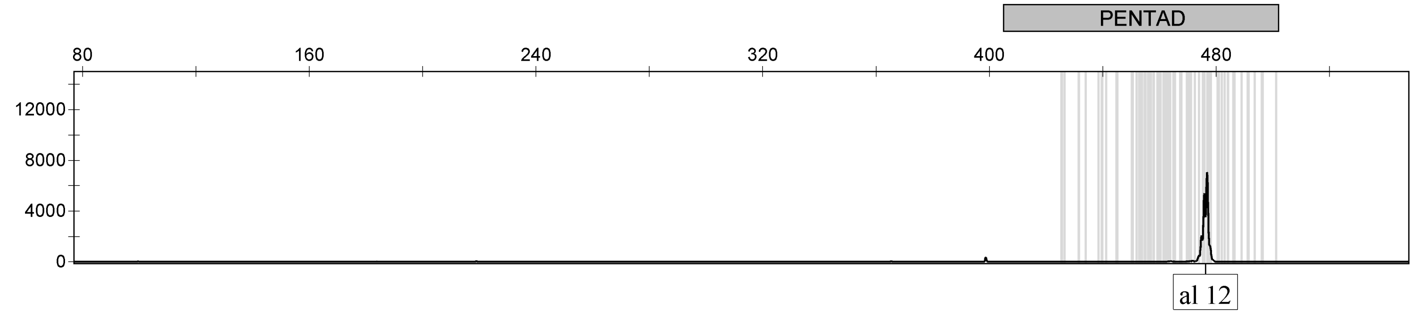

Sample File	Sample Name	Panel	OS	SQ
42_B06.fsa	3	STR Profile 3-human		

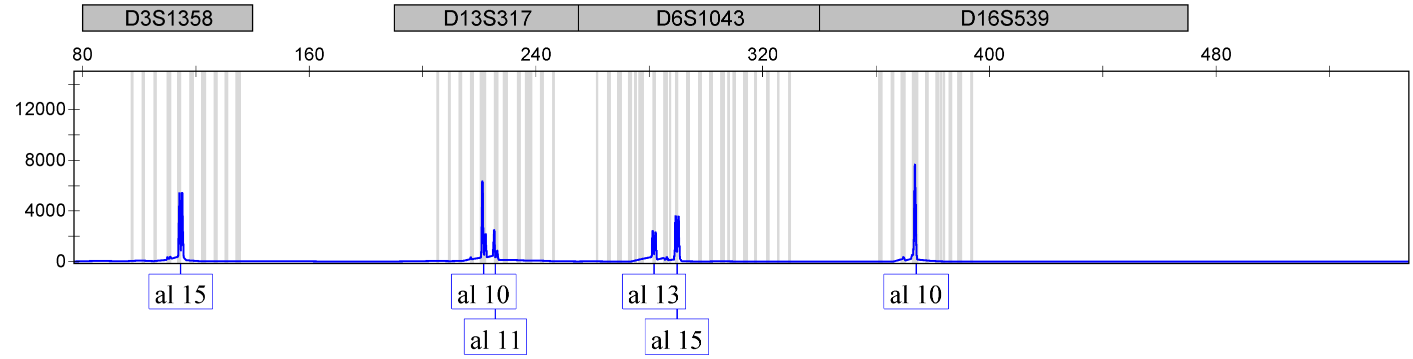

Sample File	Sample Name	Panel	OS	SQ
42_B06.fsa	3	STR Profile 3-human		

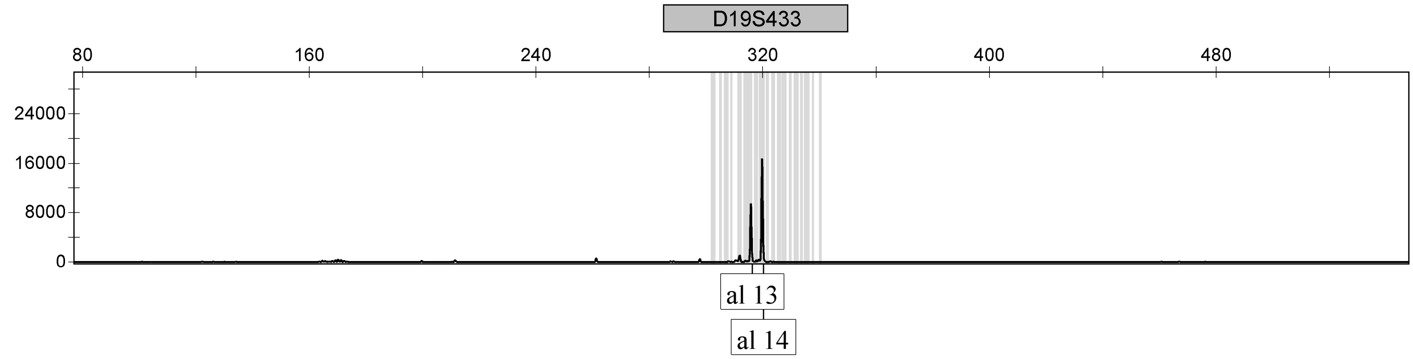

Cell Line Authentication 0920

GeneMapper 4.0

Sample File	Sample Name	Panel	OS	SQ
58_B08.fsa	3	STR Profile 4-human		

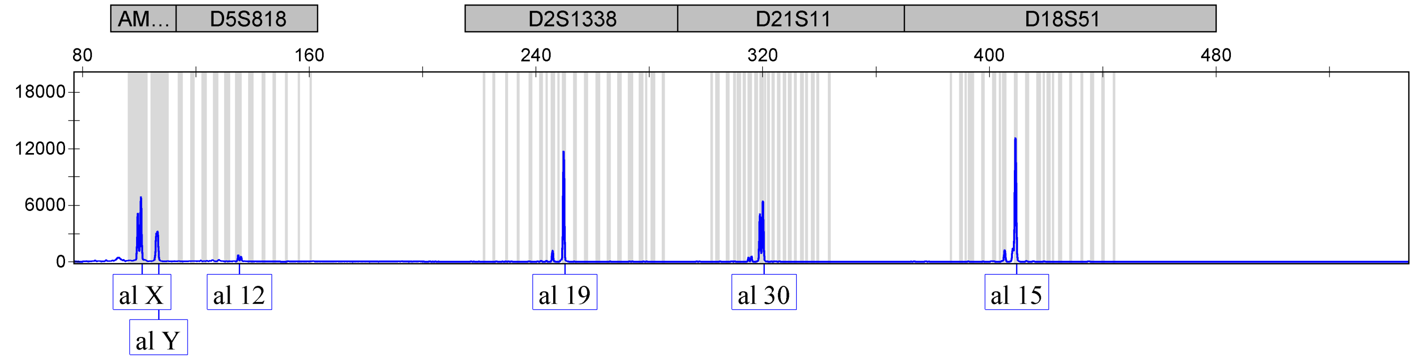

Sample File	Sample Name	Panel	OS	SQ
58_B08.fsa	3	STR Profile 4-human		

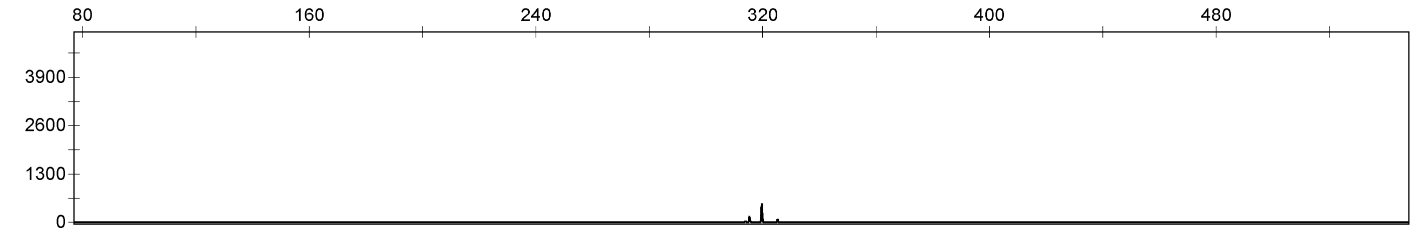

Supplement: Supplementary file 10 — HUH-6-STR Profiling [file 41419_2019_1943_MOESM10_ESM.pdf]
