# Supplementary material for: DPEP1 is a direct target of miR-193a-5p and promotes hepatoblastoma progression by PI3K/Akt/mTOR pathway
Source: Cell Death Dis. 2019 Sep 20;10(10):701. doi: 10.1038/s41419-019-1943-0 (PMC6754441; doi:10.1038/s41419-019-1943-0)

Cell Line Authentication – STR Profiling

Sample Type:Cell Line

Sample from:The First Affiliated Hospital of Zhengzhou University,
Zhengzhou 450052, China

Testing Method: STR Genotyping

Report Time: August 10, 2019

Cell Line Authentication – STR Profiling Report

Sample code

Table 1. Sample Code

Customer's code	Company Code
4	20190810-03

Sample Number:4

Sample Type: Cell line

Testing Type: STR
Sample From: The First Affiliated Hospital of Zhengzhou University

Testing Method:

DNA was extracted by a commercial kit from CORNING (AP-EMN-BL-GDNA-250G). The twenty STRs including Amelogenin locus were amplified by six multiplex PCR and separated on ABI 3730XL Genetic Analyzer. The signals were then analyzed by the software GeneMapper.

Data Interpretation:

Cell lines were authenticated using Short Tandem Repeat (STR) analysis as described in 2012 in ANSI Standard (ASN-0002) by the ATCC Standards Development Organization (SDO) and in Capes-Davis et al., Match criteria for human cell line authentication: Where do we draw the line? Int J Cancer.2013;132(11):2510-9.

Test Results:

1. Result

Table 2. Matching information on the cell lines

Sample Code	Multi-allele	Cell line matched	Cell Bank	Percentage
20190810-03-04	NO	L02	DSMZ	9/9

- **Multi-allele means some STR contain more than two loci.**

2. Sample Description

20190810-03 The DNA of the cell lines found to basic match the type of cell lines in a cell line retrieval, DSMZ database shows that cells called L02 corresponding to the cell number JCRB0401 . No multiple alleles were found in this cell line.

3. Genotyping Result

STR and Amelogenin Genotyping Results of Cell line 20190810-03						
Loci	**Sample**：L02			**Cell Bank information**：		
	Allele1	Allele2	Allele3	Allele1	Allele2	Allele3
D5S818	11	12				
D13S317	13.3	13.3				
D7S820	12	12				

D16S539	9	10				
VWA	16	16				
TH01	7	7				
AMEL	X	X				
TPOX	12	12				
CSF1PO	10	10				
D12S391	20	20				
FGA	18	21				
D2S1338	17	17				
D21S11	27	28				
D18S51	16	16				
D8S1179	12	12				
D3S1358	15	18				
D6S1043	18	18				
PENTAE	7	17				
D19S433	13	13				
PENTAD	8	15				

Others:

1. Genotyping Strategy and Site Distribution

Attached Table. Experimental Strategy and Sites

	Strategy 1	Strategy 2	Strategy 3	Strategy 4
1	TH01	TPOX	D3S1358	AMEL
2	D12S391	VWA	D13S317	D5S818
3	D7S820	D8S1179	D6S1043	D2S1338
4	CSF1PO	PENTAD	D16S539	D21S11
5	FGA		D19S433	D18S51
6	PENTAE			

The allele match algorithm compares the 8 core loci plus amelogenin only, even though alleles from all loci will be reported when available.

2. DSMZ tools was used to carry on the cell line comparison, which contains 2455 cell lines STR data from ATCC, DSMZ, JCRB ,ECACC and RIKEN databases. If the cell is not included in the above cell library, users need to compared with other databases.

Sample File	Sample Name	Panel	OS	SQ
58 B08 WeiWeiXingXiaoShi1-6XBJD7-0607.fsa	12	STR Profile 1-human		

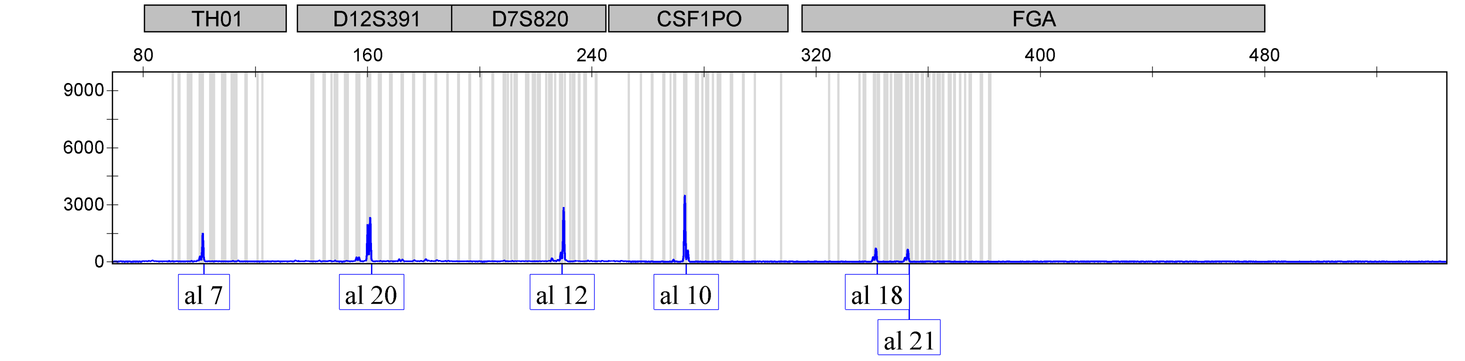

Sample File	Sample Name	Panel	OS	SQ
58 B08 WeiWeiXingXiaoShi1-6XBJD7-0607.fsa	12	STR Profile 1-human		

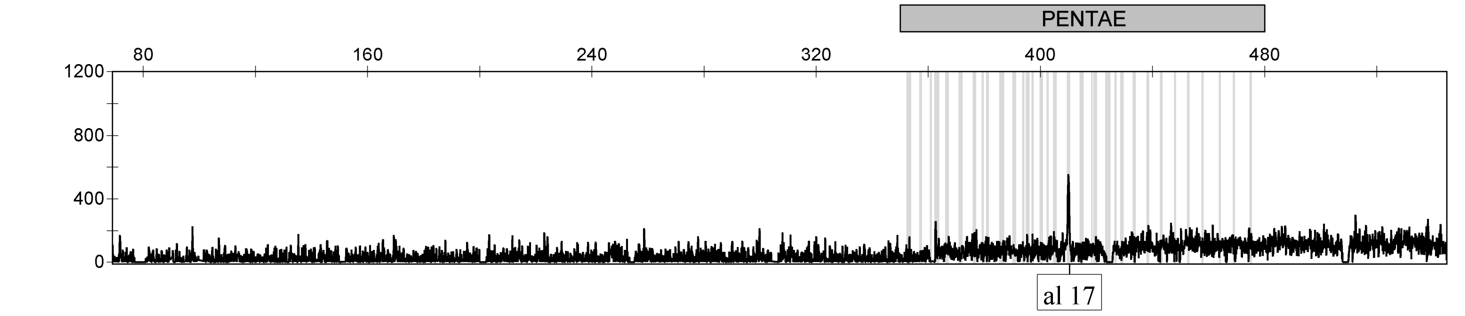

Sample File	Sample Name	Panel	OS	SQ
61 E08 WeiWeiXingXiaoShi1-6XBJD7-0607.fsa	12	STR Profile 2-human		

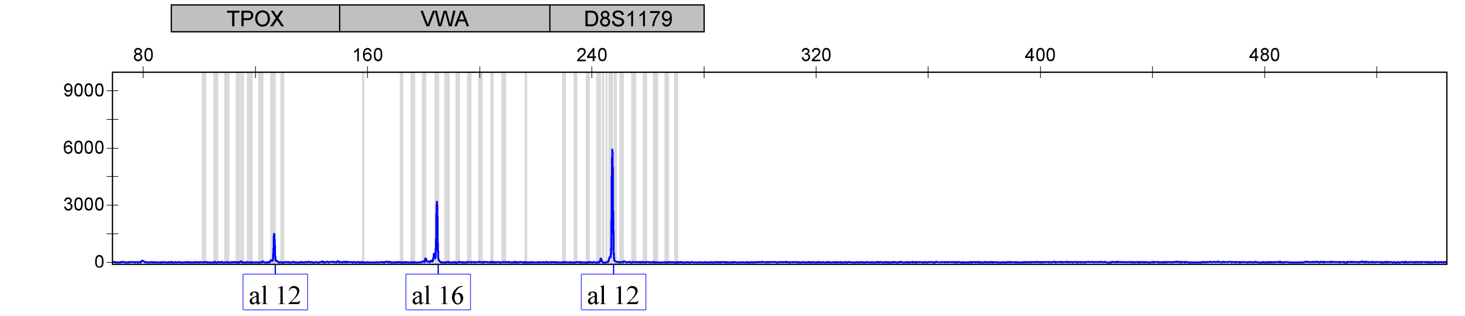

Sample File	Sample Name	Panel	OS	SQ
61 E08 WeiWeiXingXiaoShi1-6XBJD7-0607.fsa	12	STR Profile 2-human		

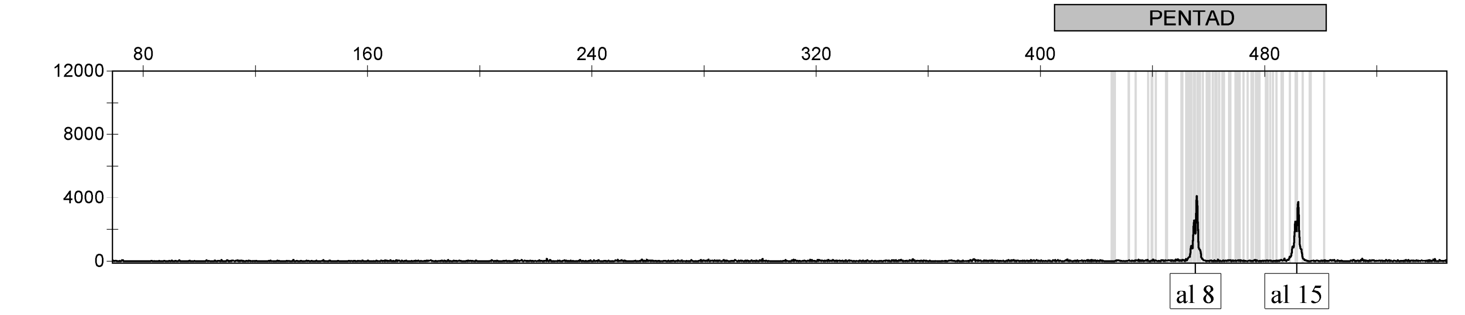

GeneMapper 4.0

Sample File	Sample Name	Panel	OS	SQ
21_E03_CellLineAuthentication-0608.fsa	12	STR Profile 3-human		

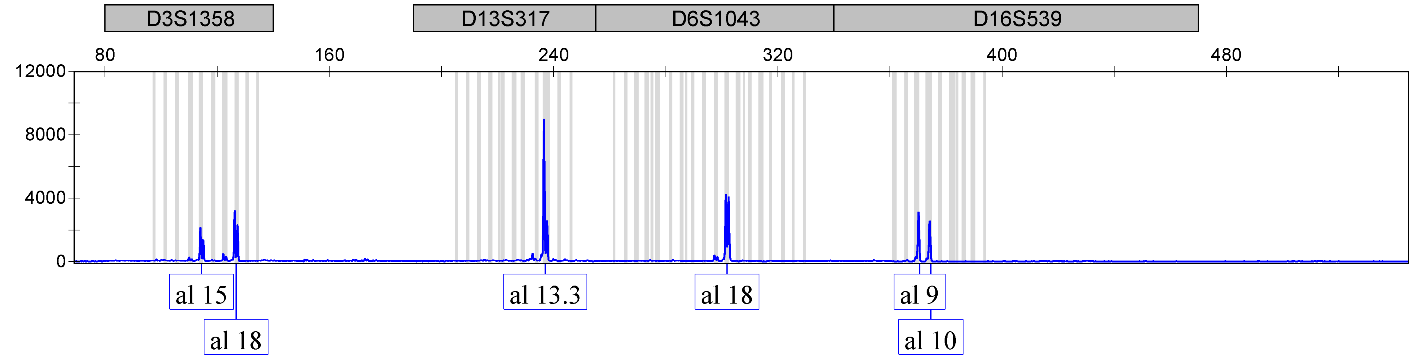

21_E03_CellLineAuthentication-0608.fsa	12	STR Profile 3-human		

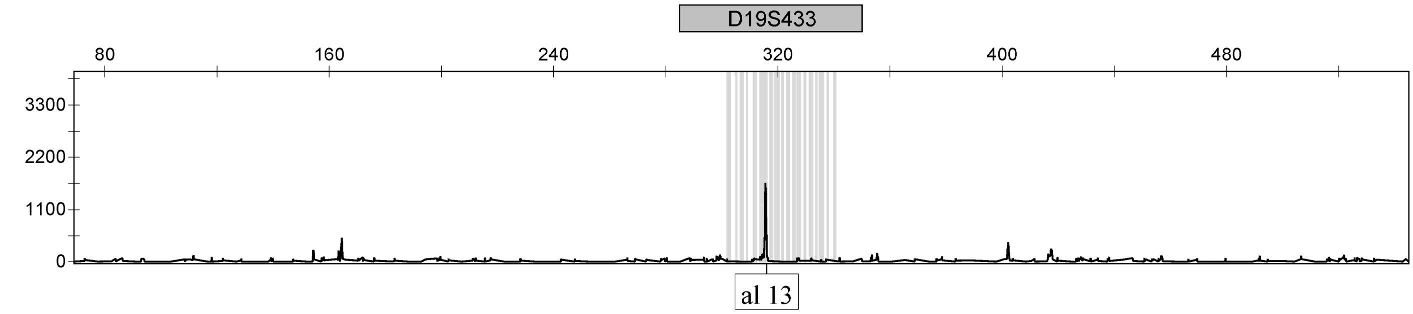

18_B03_CellLineAuthentication-0608.fsa	12	STR Profile 4-human		

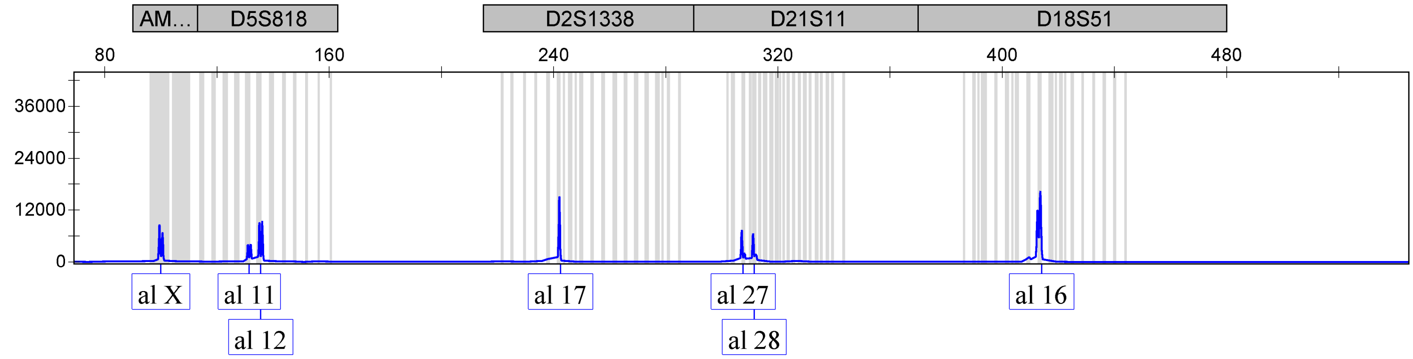

18_B03_CellLineAuthentication-0608.fsa	12	STR Profile 4-human		

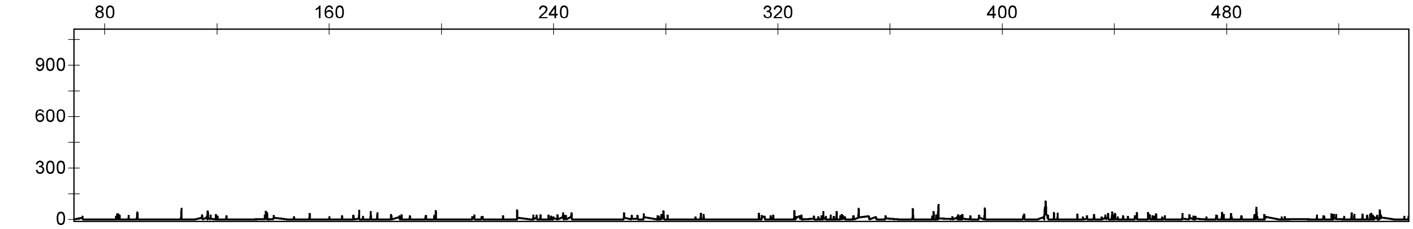

Supplement: Supplementary file 11 — L02-STR Profiling [file 41419_2019_1943_MOESM11_ESM.pdf]
